# The induction of core pluripotency master regulators in cancers defines poor clinical outcomes and treatment resistance

**DOI:** 10.1038/s41388-019-0712-y

**Published:** 2019-02-11

**Authors:** A. C. Hepburn, R. E. Steele, R. Veeratterapillay, L. Wilson, E. E. Kounatidou, A. Barnard, P. Berry, J. R. Cassidy, M. Moad, A. El-Sherif, L. Gaughan, I. G. Mills, C. N. Robson, R. Heer

**Affiliations:** 1 Northern Institute for Cancer Research, Newcastle University, Framlington Place, Newcastle upon Tyne, NE2 4HH UK; 2 Prostate Cancer UK/Movember Centre of Excellence for Prostate Cancer, Centre for Cancer Research and Cell Biology, Queen’s University of Belfast, Belfast, BT9 7AE UK; 3 Department of Urology, Freeman Hospital, The Newcastle upon Tyne Hospitals NHS Foundation Trust, Newcastle upon Tyne, NE7 7DN UK; 4 Department of Pathology, Royal Victoria Infirmary, The Newcastle upon Tyne Hospitals NHS Foundation Trust, Newcastle upon Tyne, NE1 4LP UK; 5 Nuffield Department of Surgical Sciences, University of Oxford, Oxford, OX3 9DU UK

**Keywords:** Cancer models, Prostate cancer

## Abstract

Stem cell characteristics have been associated with treatment resistance and poor prognosis across many cancer types. The ability to induce and regulate the pathways that sustain these characteristic hallmarks of lethal cancers in a novel in vitro model would greatly enhance our understanding of cancer progression and treatment resistance. In this work, we present such a model, based simply on applying standard pluripotency/embryonic stem cell media alone. Core pluripotency stem cell master regulators (OCT4, SOX2 and NANOG) along with epithelial–mesenchymal transition (EMT) markers (Snail, Slug, vimentin and N-cadherin) were induced in human prostate, breast, lung, bladder, colorectal, and renal cancer cells. RNA sequencing revealed pathways activated by pluripotency inducing culture that were shared across all cancers examined. These pathways highlight a potential core mechanism of treatment resistance. With a focus on prostate cancer, the culture-based induction of core pluripotent stem cell regulators was shown to promote survival in castrate conditions—mimicking first line treatment resistance with hormonal therapies. This acquired phenotype was shown to be mediated through the upregulation of iodothyronine deiodinase DIO2, a critical modulator of the thyroid hormone signalling pathway. Subsequent inhibition of DIO2 was shown to supress expression of prostate specific antigen, the cardinal clinical biomarker of prostate cancer progression and highlighted a novel target for clinical translation in this otherwise fatal disease. This study identifies a new and widely accessible simple preclinical model to recreate and explore underpinning pathways of lethal disease and treatment resistance.

## Introduction

One of the most daunting clinical challenges is the management of treatment-resistant cancer that leads to incurable lethal disease. Preclinical studies have demonstrated significant advances in our ability to study aggressive cancer in vitro. More recently, 2D and 3D models have yielded important mechanistic insight and therapeutic targets in aggressive cancers including establishment of field defining tumour organoid cultures and patient-derived xenografts [1–5]. Furthermore, gene expression studies from clinical data show that transcription signatures associated with stem cell pathways identify aggressive cancers and inform on poor outcomes to therapy in a wide range of malignancies including breast, glioma, bladder, prostate, lung and ovarian cancers [6–8]. The ability to recreate these phenotypes in vitro would allow a new approach to explore underpinning pathways of treatment resistance and the drivers behind aggressive disease.

Previously, a small number of transcription factors including octamer-binding factor 4 (OCT4/Pou5f1), SRY box-containing factor 2 (SOX2), and NANOG were identified as the master regulators of self-renewal and pluripotency of embryonic stem cells (ESCs) [9]. These factors were subsequently shown to reprogramme somatic cells into induced pluripotent stem cells (iPSCs) [10, 11] and are thought to promote lineage plasticity that allows cancer cells to adapt to growing in distinct sites of the body and to develop resistance to cancer therapy [5, 12, 13]. Additionally, core stem cell traits namely self-renewal, hyperproliferation, suppressed apoptosis, evasion of immune surveillance and plasticity are often shared by tumours and moreover co-opted expression of these stem cell master regulators has been reported in many cancers [14–18]. These discoveries suggest that regulation of ESC identity, cellular reprogramming and tumorigenesis may share common pathways controlled by the same core master regulators [19, 20]. Since reprogramming techniques for iPSC generation have demonstrated that stem cell culture contains environmental drivers that induce and maintain pluripotent factors within associated cells [21], we explored using this approach to generate a new in vitro model of inducing stem cell characteristics in human cancer cells.

In this study, we confirm that clinical expression of pluripotent factors OCT4, SOX2 and NANOG is associated with treatment resistance and lethal cancers. Then, describing a new model to induce these factors in vitro, we reveal treatment resistance pathways across common cancers and, as an example in prostate cancer, we uncover a new treatment target for castration-resistant disease, the thyroid hormone (TH) pathway.

## Results

### Expression of pluripotency master regulators identifies aggressive cancers and disease resistance

**Fig. 1:**
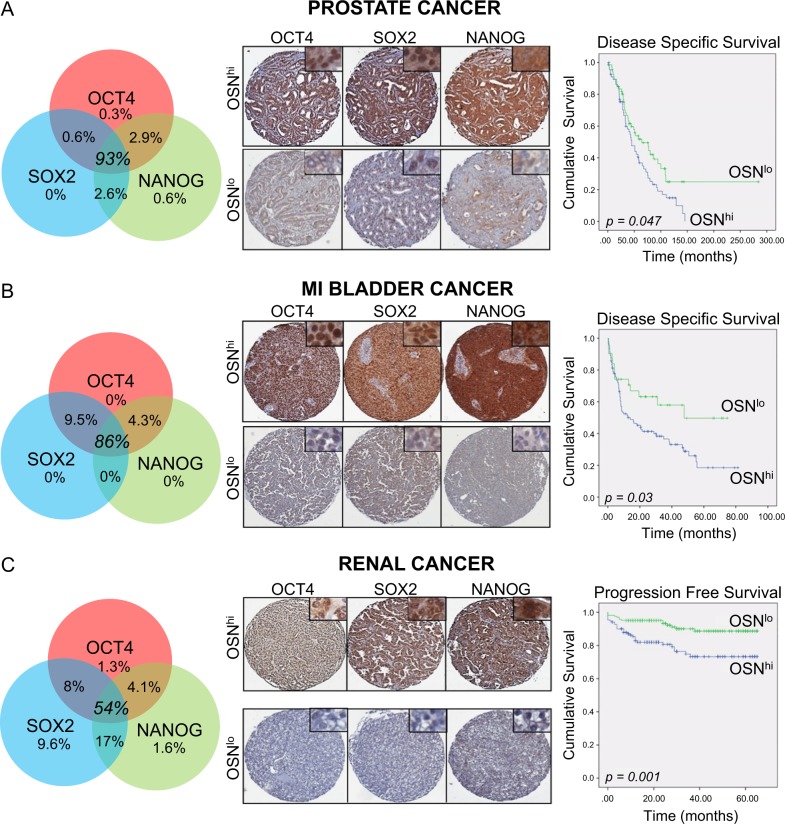
Expression of pluripotency master regulators identifies aggressive cancers and disease resistance. **a** Immunohistochemical analysis of tissue microarrays for prostate cancer demonstrating frequency of OCT4, SOX2 and NANOG expression (left panel), illustrative tissue cores of prostate cancer stained for OCT4, SOX2 and NANOG (OSN) representative of high (OSN^hi^) and low (OSN^lo^) levels of expression (middle panel) and correlation of OSN sum score with disease-specific survival (DSS) by Kaplan–Meier analysis (right panel). **b** Same as **a** but for muscle-invasive bladder cancer (MIBC). **c** Same as **b** but for renal cancer. Note correlation of OSN sum score with Progression Free Survival (PFS) by Kaplan–Meier analysis (right panel)

As aggressive cancers have been previously shown to be marked by the expression of a large network of stem cell genes [6, 7], it was hypothesised they could alternatively be defined, by proxy, by the expression of just a small number of core pluripotent master regulators. To investigate if these master regulators could act as a core signature for clinically aggressive cancers, we explored an *OCT4/SOX2/NANOG* (OSN) expression signature in a large cohort of clinical cancers (*n* = 884), comprising of prostate (*n* = 275), bladder (*n* = 292) and renal cancers (*n* = 317) (Fig. 1). Specifically, when comparing prostate cancer to ageing prostate controls, benign prostatic hyperplasia (BPH), we demonstrated significant upregulation of OSN (*n* = 67 cancers, sum OSN score 4.7 ± 2.4 vs *n* = 34 BPH, sum OSN score 3.03 ± 1.1) (Fig. S2A; *t-*test, *p* = 0.0002). This treatment naïve cohort contained locally advanced or metastatic prostate cancers that were subsequently treated with androgen deprivation therapy (ADT) and we showed that 93% of patients co-expressed all three factors (OSN), indicating the co-operative recruitment of master regulators promoting a pluripotent phenotype (Fig. 1a, left and middle panel). We went on to demonstrate that high levels of OSN expression were associated with a worse prognosis with Kaplan–Meier analysis indicating that OSN^hi^ prostate cancer patients (*n* = 69) had shorter disease specific survival (DSS) than OSN^lo^ patients (*n* = 67) (log-rank test, *p* = 0.047) (Fig. 1a, right panel). A similar pattern was demonstrated in the immunostaining of life-threatening muscle-invasive bladder cancers (MIBC) for OSN which showed 86% of patients expressed all three factors and OSN^hi^ patients (*n* = 63) demonstrated shorter DSS than OSN^lo^ patients (*n* = 59) (Fig. 1b; *t-*test, *p* = 0.03). We then explored OSN expression in non-muscle-invasive bladder cancer (NMIBC) (Fig. S2B-C), where the clinical problem is the risk of recurrence and in those patients with high-grade tumours the risk of progression to muscle-invasive disease. We showed that high levels of OSN expression in NMIBC patients was associated with higher rates of recurrent disease (33.3%) and features that leave patients at high risk of progression to life-threatening muscle-invasive disease characterised by high histological grade or early invasive histological stage into the lamina propria invasion (TNM T-stage 1) (Fig. S2D; OSN^hi^ = 110 and OSN^lo^ = 60, *χ*^2^-test, *p* = 0.02). Furthermore, the expression of high levels of OSN in NMIBC was associated with poor response to intravesical chemotherapy (Fig. S2E). In a similar fashion, the immunostaining of renal cell cancer (RCC) patients showed co-expression of all three factors, albeit at lower levels (54%), and that high level of OSN expression was associated with (*n* = 142) shorter progression free survival (PFS) compared to OSN^lo^ patients (*n* = 172) (Fig. 1c; *t-*test, *p* = 0.001). Additionally, high level of OSN expression was also associated with higher tumour stage (Fig. S2F; OSN^hi^ = 142 and OSN^lo^ = 175, *χ*^2^-test, *p* = 0.04). Collectively, these findings corroborate the association of stem cell pathways signatures with aggressive disease and that our data specifically demonstrates that master regulators of stem cell pluripotency (OSN) correlates with poor survival and treatment resistance in cancer.

### Pluripotent stem cell factors can be induced in vitro and characterises aggressive disease

**Fig. 2:**
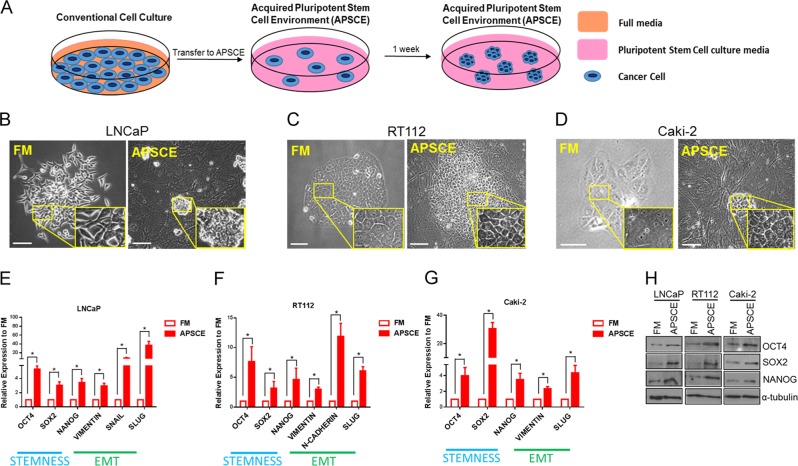
A preclinical model to recreate a stem cell-like aggressive cancer phenotype. **a** Schematic of culture details of generation of Acquired Pluripotent Stem Cell Environment (APSCE). Briefly, cancer cells were transferred from culture in their regular serum supplemented medium (FM) to culture conditions used for human embryonic stem cells (ESCs) and induced pluripotent stem cells (iPSCs) as described in ‘Materials and methods’. Following culture of cancer cells in APSCE, tight colonies consisting of smaller cells, morphologically similar to that of ESCs and iPSCs, were observed. **b**–**d** Prostate (LNCaP), bladder (RT112) and renal (Caki-2) cancer cell growth in serum supplemented medium (FM) and APSCE after 7 days. Of note, for cells cultured in APSCE, feeder cells were used though no MACS selection was performed. **e**–**g** Stemness (OCT4, SOX2, NANOG) and mesenchymal (vimentin, Snail, Slug, N-cadherin) gene expression was measured by quantitative PCR (qPCR) following culture in FM vs APSCE in LNCaP, RT112 and Caki-2 cells. Data represents at least three independent experiments ± SEM (*denotes *p*-value < 0.05). Of note, EMT characterisation studies were carried out without feeders in APSCE to avoid any mesenchymal cell contamination. **h** Western blot analysis demonstrating OCT4, SOX2 and NANOG protein expression following culture in FM and APSCE in LNCaP, RT112 and Caki-2 cells, using α-tubulin as loading control

Since reprogramming techniques for iPSC generation have demonstrated that stem cell culture can generate environmental drivers that induce pluripotent factors [21], we explored using this approach an in vitro model of inducing pluripotent stem cell characteristics. We termed this culture environment ‘Acquired Pluripotent Stem Cell Environment (APSCE)’ and comparisons were made to culture in conventional serum supplemented “full media” (FM) (Fig. 2a). Culture of prostate (LNCaP), bladder (RT112) and renal (Caki-2) cancer cells in APSCE resulted in tight colonies consisting of smaller cells, morphologically similar to that of ESCs and iPSCs [22] (Fig. 2b–d). These characteristic morphological changes were also associated with stem cell gene expressions. We confirmed APSCE significantly induced an upregulation in the expression of OSN in prostate, bladder and renal cancer cells (Fig. 2e–h). As plasticity is modulated by OSN, and in turn plasticity of cancers can be characterised by the ability to undergo EMT, a process that is known to be associated with aggressive metastatic disease [23–25], we investigated whether culture in APSCE could also induce EMT. A panel of markers was investigated including E-cadherin, N-cadherin, Slug, Snail and vimentin. Of note, it has been shown that EMT markers have different roles in different cancers and therefore we investigated expression of genes representative of EMT induction specific to the cell line [26–28]. Indeed, an upregulation in the expression of mesenchymal markers Snail, Slug, vimentin and N-cadherin was observed in APSCE (Fig. 2e–g). Additionally, pluripotent stem cell-like morphology, induction of OSN and EMT expressions, was also confirmed in breast (MCF7), colon (HCT116) and lung (H661) cancer cells cultured in APSCE (Fig. S3).

### Pluripotent factors drive a core regulatory transcriptome in cancer

**Fig. 3:**
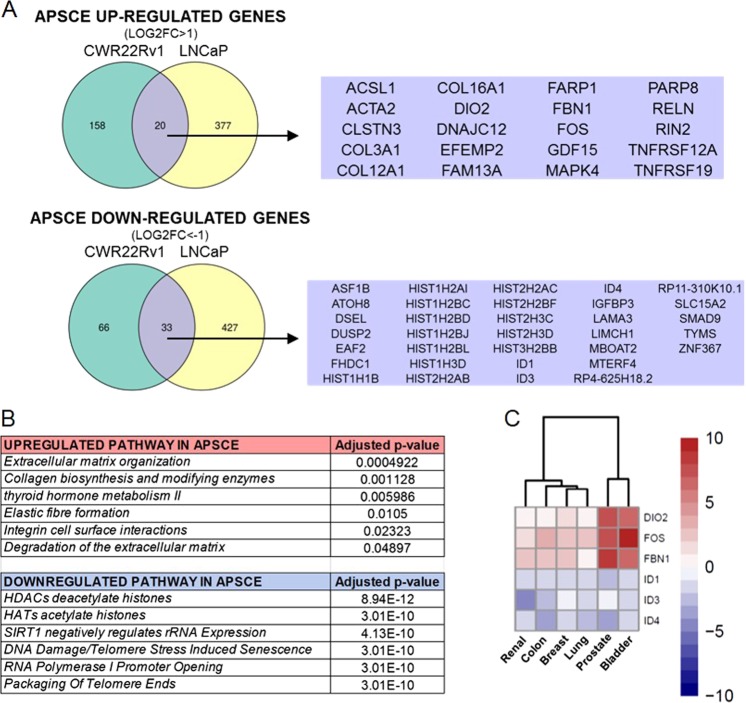
Pluripotent factors drive a core regulatory transcriptome in cancer. **a** Venn diagrams of genes demonstrating ≥2-fold up- and downregulation following culture in APSCE in prostate cancer cells (LNCaP and CWR22Rv1). The overlapping up- and downregulated genes in prostate cancer cells are noted. **b** Table of pathways most significantly altered following culture of prostate cancer cells (LNCaP and CWR22Rv1) in APSCE. **c** Heatmap demonstrating transcript expression of core regulatory signature in APSCE compared to culture in FM in prostate (LNCaP), bladder (RT112), renal (Caki-2), breast (MCF7), colon (HCT116) and lung (H661) cancer cells. Measurements made by real time PCR. Data represents at least three independent experiments and is Log2 transformed

To achieve a greater insight into the pathways recruited during OSN induction, whole transcriptome analysis using RNA sequencing was performed in prostate cancer cells, LNCaP and CWR22Rv1. Following culture in APSCE, 1081 genes were differentially expressed (adj. *p* value < 0.01) by at least twofold compared to culture in FM (Fig. 3a). Data analysis using HumanCyc and Reactome revealed deregulation of pathways in APSCE including thyroid hormone metabolism, extracellular matrix organisation and degradation, collagen biosynthesis, integrin cell surface interactions, histone modifications (Fig. 3b). As an external validation set, RNA sequencing was also performed in bladder cancer cells, RT112, and following culture in APSCE 851 genes were differentially expressed (adj. *p* value < 0.01) by at least twofold compared to culture in FM (Fig. S4A). Common characteristics that were significantly altered in APSCE of both prostate and bladder cancer cells were identified, including shared metabolism of lipids and lipoproteins pathways (including genes involved in cholesterol biosynthesis and SREBP1 signalling) (Fig. S4B, *p* < 0.05) and a shared regulatory signature of differentially expressed genes that included COL3A1 (collagen biosynthesis), COL16A1 (extracellular matrix organisation and integrin cell surface interactions), DIO2 (thyroid hormone pathway), FBN1 (extracellular matrix organisation), FOS, ID1, ID3, ID4 and IGFBP3 (Insulin growth factor pathway). (Fig. S4A). We went on to validate this core regulatory signature by confirming upregulation or downregulation of these transcripts in prostate, bladder, renal, breast, colon and lung cancer cells (Fig. 3c).

### Pluripotent stem cell factors are associated with clinical treatment resistance

**Fig. 4:**
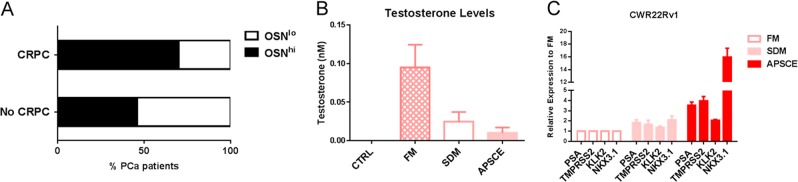
APSCE recreated phenotypes associated with clinical treatment resistance in castration-resistant prostate cancer. **a** Comparison of OSN expression and development of castration-resistant prostate cancer (CRPC) (*t-*test, *p* = 0.0016). **b** Measurement of testosterone (T) levels in FM, steroid-depleted medium (SDM) and APSCE medium including in water (CTRL). **c** Expression of PSA, KLK2, TMPRSS2 and NKX3.1 was measured by qPCR in CWR22Rv1 cells cultured in FM, SDM and APSCE. Data is represented as fold change of FM experimental arm. Data represents at least three independent experiments ± SEM

Poor outcomes in cancer treatment reflects either inherent aggressiveness of the cancer and/or acquired resistance to treatment. Specifically in prostate cancer, androgens functioning through the androgen receptor (AR) are central to both initiation and disease progression [29]. Thus, androgen deprivation therapy (ADT) is the mainstay for advanced prostate cancer patients, but in time there is inevitable disease progression and development of lethal treatment-resistant prostate cancer. Using the clinical biochemical measure of prostate specific antigen (PSA) for treatment response, 70% of OSN^hi^ prostate patients went on to develop resistance to ADT in 1.9 years (*n* = 58) compared to 54% of OSN^lo^ patients (*n* = 55) (Fig. 4a; *t-*test, *p* = 0.0016). Also, OSN^lo^ patients had a lower PSA nadir and showed a greater fold reduction (PSA at presentation/PSA nadir), indicative of a better response to ADT (Fig. S5A).

Having confirmed castration levels of testosterone in the APSCE to be comparable with the conventional model of ADT in steroid-depleted medium (SDM) [30, 31] (Fig. 4b), we noted a paradoxical upregulation of AR target gene expression following culture of castration-resistant prostate cancer cells CWR22Rv1 in APSCE (Fig. 4c, Fig. S5B). Specifically, we confirmed functional activity of AR through increased expression of downstream readouts of PSA, TMPRSS2, KLK2 and NKX3.1 transcripts. A similar induction was also seen in classically hormone-dependent LNCaP cells when cultured in APSCE, which were now able to be maintained without becoming abortive colonies in the absence of androgens (Fig. S5C-D). These behaviours are the hallmarks of castration-resistant prostate cancer underpinned by bypass signalling pathways characterised through various mechanisms of aberrant AR activation [32].

### Acquired pluripotent pathways drive castration-resistant prostate cancer independent of conventional AR signalling

**Fig. 5:**
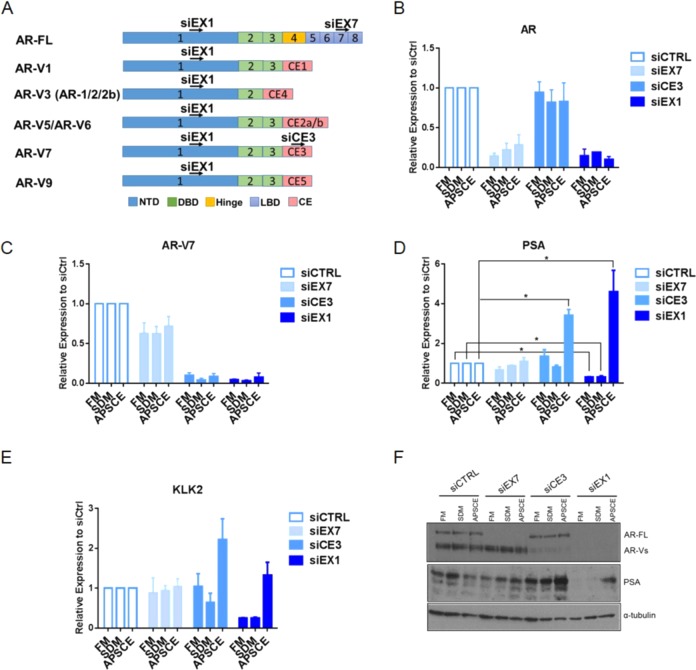
Acquired pluripotent pathways drive castration-resistant prostate cancer independent of conventional AR signalling. **a** Diagram illustrating the exons of the androgen receptor (AR) and variants (AR-Vs) that siEX1, siEX7 and siCE3 target. NTD, N-terminal domain; DBD, DNA-binding domain; LBD, ligand-binding domain; CE, Cryptic exon. **b** Expression of AR was measured by qPCR following knockdown of AR-FL (siEX7), AR-V7 (siCE3) and all AR (siEX1) in CWR22Rv1 cells cultured in FM, SDM and APSCE. Data is represented as fold change of siCTRL experimental arm. Data represents at least three independent experiments ± SEM. **c** Same as in **b** but measurement of AR-V7 expression. **d** Same as in **c** but measurement of PSA expression (*denotes *p*-value < 0.05). **e** Same as in **c** but measurement of KLK2 expression. **f** Resultant lysates from samples in **b**, **c**, **d** and **e** were subjected to western blot analysis to measure AR and PSA expression. AR-N20 antibody was used to detect both AR-FL and AR-Vs and α-tubulin was used as loading control

Recently, multiple AR splice variants (AR-Vs) that lack the ligand-binding domain (LBD) have been described to be constitutively active in the absence of androgen ligand and contribute to the development of castration-resistant prostate cancer (CRPC) [33, 34]. Reports suggest AR-Vs can regulate canonical AR target genes as well as their own distinct transcriptome and as they lack the LBD they evade inhibition by antiandrogens [35, 36]. This was validated in the CWR22Rv1 model expressing both full-length AR (AR-FL) and AR-Vs, whereby AR target genes were still expressed following knockdown of AR-FL with siRNA targeting exon 7 (siEX7) (Fig. 5a–f). Since we found APSCE, a castrate environment, was associated with paradoxical upregulation of AR target genes, we speculated this was driven by AR-Vs. To confirm this, we assessed AR target gene expression following depletion of AR-V7, the predominant constitutively expressed variant in CWR22Rv1, with siRNA targeting cryptic exon 3 (siCE3) (Fig. 5c–f). Depletion of AR-V7 resulted in increased expression of PSA and KLK2 in APSCE suggesting regulation by low level AR-FL activity. Thus, we next assessed depletion of all AR species with siRNA against exon 1 (siEX1) to target AR-FL and AR-Vs (Fig. 5b, c and f). As anticipated, siEX1 decreased AR target gene expression in FM and SDM (Fig. 5d–f, Fig. S6A-B). Intriguingly, we still observed upregulation of PSA and KLK2 in APSCE (Fig. 5d–f). Cytoplasmic/nuclear fractionation studies demonstrated absence of AR from the respective cellular compartments and no binding to chromatin following siEX1, providing further confirmation of effective AR knockdown (Fig. S6C). Additionally, treatment with ENZ following siEX1, to further inhibit possible low levels of AR-FL below our sensitivity for detection, also did not reduce PSA expression below basal level (Fig. S6D). Taken together, these findings suggest alternative non-AR pathways are being recruited in this environment to regulate AR target gene expression. However, we did observe decreased TMPRSS2 and NKX3.1 levels following depletion of all AR and AR-V7 alone (Fig. S6A-B), demonstrating the differential effect of APSCE on downstream reporters of AR activity and that AR signalling may still be an active component in this environment.

Because *OCT4, SOX2* and *NANOG* are considered to be master regulators of the pluripotent state of ESCs and iPSCs and their induction was shown in APSCE, we asked whether these factors were required for the stem cell-like inductions. We performed knockdown studies in prostate cancer cells using siRNA against all three factors (siOSN) and confirmed downregulation of the cardinal biomarkers of prostate cancer progression (PSA and KLK2) in FM (Fig. S7A). APSCE media had much higher levels of induced OSN and it was not possible to achieve knockdown with similar concentrations of siRNA in this background of competing upregulation from the environment (Fig. S7B-D).

**Fig. 6:**
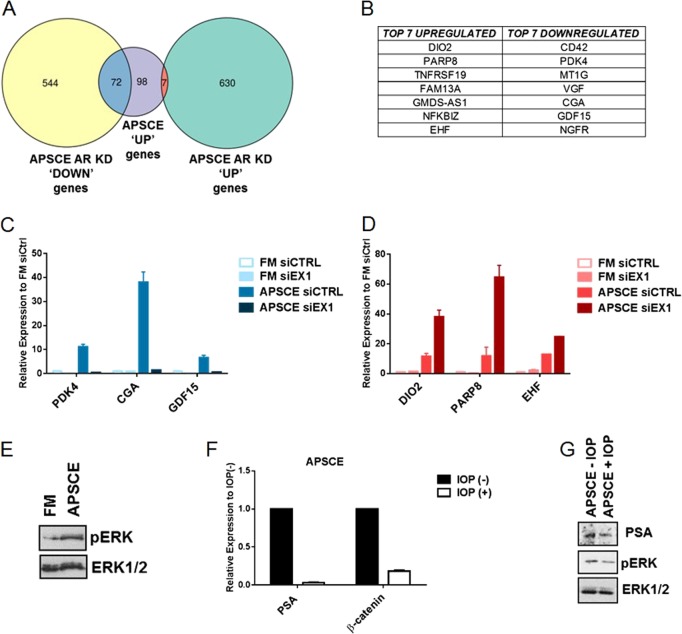
APSCE identifies the thyroid hormone signalling network as a target to overcome castration resistance in prostate cancer. **a** Venn diagram demonstrating genes upregulated in APSCE and up- or downregulated following AR depletion with siEX1 in APSCE in CWR22Rv1 cells. **b** Top seven up- and downregulated genes in APSCE and following AR depletion with siEX1 in APSCE in CWR22Rv1 cells (where CGA, Chorionic Gonadotrophin subunit Alpha). **c** Validation of downregulated genes in the APSCE and following AR depletion with siEX1 by qPCR analysis of CWR22Rv1 cells after AR depletion with siEX1 in FM and APSCE. Data is represented as fold change of FM siCTRL experimental arm. Data represents at least three independent experiments ± SEM. **d** Same as in **c** but validation for upregulated genes. **e** Western blot analysis measuring p-ERK expression in CWR22Rv1 cells cultured in FM and APSCE. Total ERK (ERK1/2) was used as loading control. **f** Effect of DIO2 inhibitor, iopanoic acid (IOP, 50 µM) for 24 h on PSA and β-catenin expression in CWR22RV1 cells in APSCE. **g** Western blot analysis measuring PSA and p-ERK expression following treatment of CWR22Rv1 cells with IOP (50 µM) for 24 h in APSCE. Total ERK (ERK1/2) was used as loading control

Next, we proceeded to identify those non-AR pathways recruited in APSCE by performing whole transcriptome analysis using RNA sequencing (RNA-Seq) in CWR22Rv1 cells following knockdown with siEX1 in FM and APSCE. The observed changes in AR regulated target gene expression in APSCE were similarly confirmed (Fig. S8A). Knockdown with siEX1 in APSCE resulted in 1253 genes significantly altered (adj. p value < 0.01) by at least twofold, with 637 upregulated and 616 downregulated genes (Fig. 6a, Fig. S8B). Forty-five percent of genes upregulated in APSCE in CWR22Rv1 cells were significantly affected by knockdown with siEX1. Interestingly, DIO2 was upregulated following siEX1 knockdown (Fig. 6b), also shown to be upregulated in prostate and bladder cancer APSCE (Fig. 3a and Fig. S4A). Furthermore, PARP8, TNFRSF19, FAM13A and GDF15 were also significantly altered following siEX1 knockdown (Fig. 6b), also seen to be upregulated in APSCE in prostate LNCaP cells (Fig. 3a). As shown in Fig. 6c, d, the identified genes were indeed upregulated or downregulated in APSCE following siEX1 knockdown. Importantly, DIO2, an iodothyronine deiodinase, plays a critical role in modulating thyroid hormone (TH) signalling. Deiodinase 2 (DIO2) catalyses the conversion of the prohormone thyroxine (T4) to the biologically active TH, triiodothyronine (T3), thus enhancing thyroid hormone signalling [37]. TH functions, important for growth, development and metabolism, are mediated through nuclear thyroid hormone receptors controlling the expression of target genes directly or indirectly through activation of ERK1/2 MAPK pathway, also known promoter of aggressive phenotypes in prostate cancer [38]. Indeed, increased pERK levels were observed in APSCE (Fig. 6e). Furthermore, knockdown of DIO2 in APSCE in CWR22Rv1 cells resulted in decreased PSA expression (Fig. S9A-B). Tissue expression of DIO2 also demonstrated utility across a prostate cancer cohort based on hormone treated prostate cancer patients (Fig. S9C). Additionally, treatment with DIO2 inhibitor, iopanoic acid (IOP, 50 µM), at a concentration reported to inhibit iodothyronine binding to the nuclear TH receptor [39], resulted in decreased PSA and TH targets, β-catenin and pERK, expression (Fig. 6f, g). Thus, these data implicate this pathway in castration resistance in prostate cancer.

## Discussion

Identification of the stem cell regulatory pathways underpinning treatment resistance that leads to incurable lethal disease could identify actionable therapeutic targets to overcome this major clinical problem. Here, we describe a preclinical model that recapitulates the aggressive and stem cell-like phenotypes seen in cancer using a novel application of pluripotency enhancing culture technology. This model exhibited inducible upregulation of a stem cell gene expression signature characterised by key regulators of pluripotency and associated with aggressiveness and poor cancer survival in prostate, breast, bladder, lung, colorectal and renal cancer. A shared regulatory transcriptome activated in this model across a number of cancers was also identified, highlighting a common utility targeted treatment. Using prostate cancer as an exemplar, we showed that acquisition of pluripotent master regulators drove castration resistance independent from conventional AR related signalling and uncovered the TH pathway as a new treatment target for castration-resistant disease.

Our confirmation of the clinical expression of OSN in local tumours and its correlation with lethal metastatic disease, points to a mechanism within primary tumour biology that potentially promotes metastatic initiation. Cancer cells expressing stem cell signatures have been detected in blood and subsequently demonstrated as having the capacity to generate metastasis [40, 41]. The microenvironment and environmental cues, such as hypoxia, can induce stem cell signatures resulting in the acquisition of invasive and migratory phenotypes [12, 42, 43]. Moreover, pluripotency factors have been shown to be mediators of plasticity of key microenvironmental cell players and of enhanced ECM production leading to metastatic niche formation and metastasis [44].

In APSCE, induction of pluripotent stem cells factors was associated with reprogramming of AR regulated gene expression independent of AR and recruitment of alternative pathways. Recently, a growing body of evidence has reported a role for NANOG and SOX2 in promoting castration resistance [5, 13, 45, 46]. Notably, NANOG was demonstrated to reprogramme prostate cancer cells by repressing the AR signalling axis and activating its own distinct transcriptional programme as well as engaging that of others such as MYC, leading to acquisition of a castration-resistant stem cell-like state [13]. Moreover, SOX2 was identified as a mediator of lineage plasticity, specifically neuroendocrine transdifferentiation, which was associated with decreased AR dependency and acquisition of antiandrogen enzalutamide resistance [5]. There is now increasing recognition of phenotypic shifts occurring in metastatic cancers following treatment with AR antagonists resulting in the development of highly lethal, drug-resistant castration-resistant disease such as neuroendocrine (NE) prostate cancer characterised by non-reliance on AR. Furthermore, models of AR-null phenotypes have demonstrated sensitivity to blockade of AR bypass pathways such as FGF/MAPK [47]. Our model did not recreate NE differentiation and for those studies other models are more appropriate [48]. It also remains unclear what drives PSA expression in this model, and other prime candidates such as glucocorticoid receptor and progesterone receptor nor their canonical downstream reporters were enriched in the RNA sequencing analysis in APSCE. Androgen hypersensitivity may also be a possible mechanism of resistance in APSCE since decreased levels of other downstream reporters of AR activity (TMPRSS2 and NKX3.1) were observed following depletion of all AR forms (full-length and spliced). Irrespective of these mechanisms, the identified downstream actionable targets still remain of therapeutic interest. Although a contribution of OCT4, SOX2 and NANOG to AR target gene expression was observed in androgen-dependent conditions, further exploration in APSCE was not possible due to inability to knockdown all three factors simultaneously or even individually in this environment. OCT4, SOX2 and NANOG not only function together to maintain the pluripotent state of ESCs but they also positively regulate their own promoters as well as each other thus forming an interconnected auto- and cross-regulatory loop [9].

We showed that targeting iodothyronine deiodinase *DIO2*, a critical modulator of the TH signalling pathway, presents a new approach to overcoming castration-resistant disease. Preclinical studies have shown androgens and T3 exert similar effects on cell cycle that appear to be mediated independently through differentially regulated genes [49]. Clinically, epidemiological data suggest a tumour-promoting effect for TH with T3 levels increased in men with prostate cancer and associated with higher clinical stage and risk of disease recurrence in men with localised prostate cancer [50, 51]. Furthermore, subclinical hypothyroidism measured through elevated thyroid stimulating hormone (TSH) levels demonstrated a protective effect on prostate cancer [52, 53] and overt treatment-induced hypothyroidism was associated with favourable outcome in RCC [54]. Thus, DIO2 inhibition lays foundations for a late translational/early phase clinical trial for use of antithyroid drugs, such as propylthiouracil, in men with CRPC and potentially in other cancers too. Additionally, inhibition of DIO2 has been noted to underlie the elevated plasma TSH levels associated with treatment using the widely prescribed cardiac antiarrhythmic drug amiodarone [55] and repurposing of such approved drugs could be a promising approach to overcome treatment resistance in prostate cancer. However, further understanding the role of DIO2 in prostate and other cancers requires additional investigation. Finally, the simple culture-based model we describe in this report provides an accessibility to research laboratories worldwide to similarly induce aggressive stem cell characteristics in vitro and enable the exploration of treatment resistance mechanisms and associated target discovery in other cancers.

## Materials and methods

### Clinical material

All patient tissue samples were used in accordance with approval granted by the Northumberland, Tyne and Wear NHS Strategic Health Authority Research Ethics Committee (reference 2003/11; The Freeman Hospital) and informed consent from all patients. The demographic characteristics for the prostate cancer, non-muscle-invasive bladder cancer, muscle-invasive bladder cancer and renal cancer patient samples used in this study are described in Tables S1–4. A total of 34 benign prostatic hyperplasia (BPH) samples (median age, 64; range 39–70) were also utilised.

### Immunohistochemical analysis

Immunohistochemistry was performed using tissue microarrays containing 0.6-mm cores of BPH, prostate cancer, bladder cancer, renal cancer and control tissues including breast, kidney, placenta, ovary, and liver as described [56]. Sections were immunostained with anti-OCT4 1:50 (Millipore, cat. no MABD76), anti-SOX2 1:500 (Millipore, cat. no MAB4343), anti-NANOG 1:5000 (Cell Signaling, cat. no 4893) and anti-DIO2 1:500 (Abcam, cat. no ab135711) and viewed using Aperio CS2 (Leica Biosystems). Human prostate-derived induced pluripotent stem cells, previously confirmed to express OCT4, SOX2 and NANOG, were selected as positive controls (Fig. S1A) [22]. Negative controls were prepared by incubating without the primary antibody (Fig. S1B). Immunostaining was reviewed and scored independently by two uro-pathologists that were blinded to the clinical data to give average scores of staining intensities of absent (0), weak (1), moderate (2) or strong (3). Using a previously validated approach [56, 57], a sum score for combined expression of all three factors (OCT4, SOX2 and NANOG, OSN) was calculated to compare high (OSN^hi^) and low (OSN^lo^) levels of OSN expression. Data was dichotomised at the median sum score (maximum of 9) with OSN^hi^ ≥ sum score 5 and OSN^lo^ < sum score 5.

### Immunofluorescence

Cells were fixed in 4% paraformaldehyde and permeabilised with 0.1% Triton X-100. Primary antibodies used were anti-OCT4 1:100 (Millipore), anti-SOX2 1:100 (Millipore) and anti-NANOG 1:100 (Cell Signaling). Secondary antibodies used were Alexa Fluor 488 and Alexa Fluor 546 (both at 1:500; Life Technologies). Cells were washed with 1 × PBS three times between incubations. Cells were mounted using Vectashield with DAPI mountant (Vector Laboratories) before being visualised on a confocal laser scanning microscopy system (Nikon).

### Cell culture

Human cancer cell lines LNCaP (derived from localised prostate cancer), CWR22Rv1 (derived from the human prostate cancer xenograft CWR22R), RT112 (derived from transitional cell bladder cancer), Caki-2 (derived from clear renal cell cancer), MCF7 (derived from breast cancer), HCT116 (derived from colorectal cancer) and H661 (derived from lung cancer) were obtained from the American Type Culture Collection (Manassas, VA, USA). Cell lines were authenticated by STR profiling (Newgene). All cell lines were cultured in RPMI 1640 supplemented with 10% (v/v) foetal calf serum (FCS) and 1% (v/v) L-glutamine (referred to as FM). All cells were grown at 37 °C in the presence of 5% CO_2_. All cell culture reagents were purchased from Sigma. For studies in androgen-depleted conditions, cells were cultured in RPMI 1640 supplemented with 10% (v/v) charcoal-stripped FCS (Biowest) and 1% (v/v) L-glutamine (referred to as SDM). For studies using ESC/iPSC culture conditions (referred to as Acquired Pluripotent Stem Cell Environment, APSCE), cells were seeded onto gelatin-coated plates with a layer of irradiated CF-1 mouse embryonic fibroblasts (MEFs, VhBio) in human ESC/iPSC culture medium (80% (v/v) knockout-DMEM, 20% knockout serum replacement, 1% (v/v) GlutaMAX-I Supplement, 1% (v/v) MEM nonessential amino acids (all from Gibco) and 8 ng/ml FGF2 (Miltenyi Biotec)). Cancer cells were separated from MEFs using magnetic activated cell sorting (MACS) for immunomagnetic selection of Epithelial Cell Adhesion Molecule (EpCAM/CD326) (Miltenyi Biotec) according to the manufacturer’s instructions. For morphology studies, 100 cells/well were seeded on six-well plates for both culture in FM and APSCE conditions. For cells cultured in APSCE, feeder cells were used though no MACS selection was performed to avoid any concerns about effects due to epithelial–mesenchymal transition (EMT) induction. The historically described use of feeder cells in ESC culture can be abandoned in our model as they were redundant in generating inducing stem cell characteristics. A feeder cell-free method was also employed whereby cells were seeded onto matrigel (Corning)-coated plates in mTeSR^TM^1 culture medium (Stemcell Technologies). Dihydrotestosterone (DHT) was used at a final concentration of 10 nM and enzalutamide (ENZ) was used at a final concentration of 10 μM.

### Androgen level measurement

Quantitation of testosterone was performed in selected reaction monitoring (SRM) mode. Mass transitions and optimized MS/MS parameters are given in Table S5. Analyst® software v1.4.1 (AB SCIEX) was used for SRM, peak integration, and analyte quantitation. Standard curves were prepared for testosterone in both tissue culture media and water in the range 0–20 ng/ml and the limit of detection (LOD) and lower limit of quantitation (LLOQ) were established in both matrices. The concentration of testosterone was measured in FM, SDM and APSCE media. Peak areas for these samples were quantified against the external calibration curves of testosterone (Table S6). Reverse phase chromatographic separation of testosterone was achieved using a Perkin Elmer Series 200 LC (Beaconsfield, UK) equipped with a Luna C8(2) column (3 μm; 20 × 4 mm i.d.) and SecurityGuard C18 column (4 × 3 mm) (Phenomenex, UK) maintained at 30 °C and a flow rate of 0.5 ml min^−1^ using the conditions in Table S7. An API4000 triple quadrupole LC/MS/MS (Applied Biosystems, USA) was used for analysis with electrospray ionization (ESI) performed in positive ion mode using nitrogen gas with source parameters found in Table S8.

### RNA extraction, reverse transcription and real time PCR (qPCR)

Total RNA was extracted using Ribozol™ RNA Extraction Reagent (Amresco) and reversed transcribed using Moloney murine leukaemia virus reverse transcriptase enzyme (Promega) according to the manufacturer’s instructions. Real time PCR (qPCR) was carried out using Platinum SYBR® green qPCR SuperMix-UDG (Invitrogen) in 384-well clear optical reaction plates using the ABI 7900HT qPCR system (Applied Biosystems) according to the manufacturer’s instructions. Levels of expression were normalised against housekeeping gene HPRT1. Primers sequences are described in Table S9.

### Western blotting

Cells were lysed and analysed by SDS-PAGE as previously described [56]. Antibodies used were: anti-AR 1:500 (AR-N20, Santa Cruz Biotechnology, cat. no sc-816), anti-PSA 1:1000 (DAKO, cat. no A0582), anti-OCT4 1:1000 (Millipore, cat. no MABD76), anti-SOX2 1:1000 (Millipore, cat. no MAB4343), anti-NANOG 1:1000 (Cell Signaling, cat. no 4893), anti-PARP1/2 1:5000 (Santa Cruz Biotechnology, cat. no sc-7150), anti-ERK1/2 1:500 (C-9, Santa Cruz Biotechnology, cat. no sc-514302), anti-phospho-ERK 1:500 (E-4, Santa Cruz Biotechnology, cat. no sc-7383) and anti-α-tubulin 1:4000 (Sigma, cat. no T9026). For cytoplasmic and nuclear fractionation experiments, extracts were separated and prepared using the NE-PER^®^ Nuclear and Cytoplasmic Extraction Reagents according to the manufacturer’s instructions (Thermo Scientific). The chromatin fraction was collected at the end of the extraction.

### siRNA transfections

Cells were reverse transfected with siRNA using RNAiMax (Invitrogen) according to the manufacturer’s instructions at a final concentration of 25 nM. siRNA sequences used were: Scrambled 5′-UUCUCCGAACGUGUCACGUdTdT-3′; AR Exon1 5′-GAAAUGAUUGCACUAUUGAUUdTdT-3′; AR Exon7 5′-CCAUCUUUCUGAAUGUCCUdTdT-3′; AR Exon3b 5′-GUAGUUGUGAGUAUCAUGAdTdT-3′; OCT4 5′-GUGCAGGCCCGAAAGAGAAUUdTdT-3′; SOX2 5′-CCAAGACGCUCAUGAAGAAUUdTdT-3′ and NANOG 5′-CGUGUGAAGAUGAGUGAAAUUdTdT-3′. All siRNAs were purchased from Sigma. siRNA sequence used for DIO2 was purchased from Dharmacon (product code J-011171-09-0002).

### Flow cytometry

Cells were fixed in 4% paraformaldehyde and permeabilised with 0.1% Triton X-100. Cells were labelled with anti-DIO2 1:10 (Abcam, cat. no ab135711) and secondary antibody Alexa Fluor 488 1:500 (Life Technologies). Controls included IgG isotype antibody (Dako) and secondary antibody only. Cells were analysed using Attune NxT Flow Cytometer (Thermo Fisher Scientific).

### RNA-Seq analysis

Total RNA was extracted from cells using Ribozol™ RNA Extraction Reagent (Amresco) following manufacturer’s instructions. RNA-Seq library construction and sequencing was performed at Otogenetics Corporation (Atlanta, USA) according to standard protocols. Resulting RNA-Seq fastq reads were aligned to Hg19 (GRCh37) using STAR [58] and mapped to genes using HTSeq counts (http://htseq.readthedocs.io/en/master/count.html). Differential expression analysis was performed on triplicate samples using DESeq2 [59]. Genes with an adjusted *p*-value ≤ 0.01 were taken forward for fold-change analysis. All heatmaps were generated using R3.4.2. Venn diagrams of overlapping gene lists were created using the Intervene Tool [60]. Data was deposited in GEO (GSE117193).

### Statistical analysis

For real time PCR studies, two-tailed paired *t*-test was used to determine statistical significance at a level of *p* < 0.05. Patient survival was analysed using the Kaplan–Meier method with *t*-test and log-rank and testing, and multivariate analysis was performed using the Cox proportional hazards model. All tests were undertaken using SPSS version 11.0 computer software (SPSS, Inc.). All tests were two-sided and a *p* value of <0.05 was taken to indicate statistical significance.

## Supplementary information

SI DATA
