# Correction: The induction of core pluripotency master regulators in cancers defines poor clinical outcomes and treatment resistance

**DOI:** 10.1038/s41388-019-0826-2

**Published:** 2019-05-08

**Authors:** A. C. Hepburn, R. E. Steele, R. Veeratterapillay, L. Wilson, E. E. Kounatidou, A. Barnard, P. Berry, J. R. Cassidy, M. Moad, A. El-Sherif, L. Gaughan, I. G. Mills, C. N. Robson, R. Heer

**Affiliations:** 1 Northern Institute for Cancer Research, Newcastle University, Framlington Place, Newcastle upon Tyne, NE2 4HH UK; 2 Prostate Cancer UK/Movember Centre of Excellence for Prostate Cancer, Centre for Cancer Research and Cell Biology, Queen’s University of Belfast, Belfast, BT9 7AE UK; 3 Department of Urology, Freeman Hospital, The Newcastle upon Tyne Hospitals NHS Foundation Trust, Newcastle upon Tyne, NE7 7DN UK; 4 Department of Pathology, Royal Victoria Infirmary, The Newcastle upon Tyne Hospitals NHS Foundation Trust, Newcastle upon Tyne, NE1 4LP UK; 5 Nuffield Department of Surgical Sciences, University of Oxford, Oxford, OX3 9DU UK

**Correction to:** **Oncogene**

10.1038/s41388-019-0712-y; published online 11 February 2019.

The original version of this article contained an error in Fig. 5a where the colours of the labels representing the Hinge and LBD of the AR were incorrect and did not match the corresponding exons. The corrected version of this Figure now appears in the article. The conclusions of this paper were not affected. The authors apologise for this error and any confusion caused.

