# Supplementary material for: The induction of core pluripotency master regulators in cancers defines poor clinical outcomes and treatment resistance
Source: Oncogene. 2019 Feb 11;38(22):4412–24. doi: 10.1038/s41388-019-0712-y (PMC6546609; doi:10.1038/s41388-019-0712-y)

**Supplementary Information**

B.

**
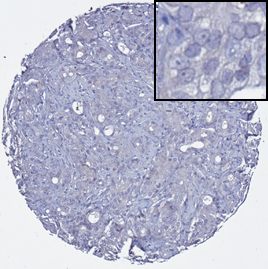
**


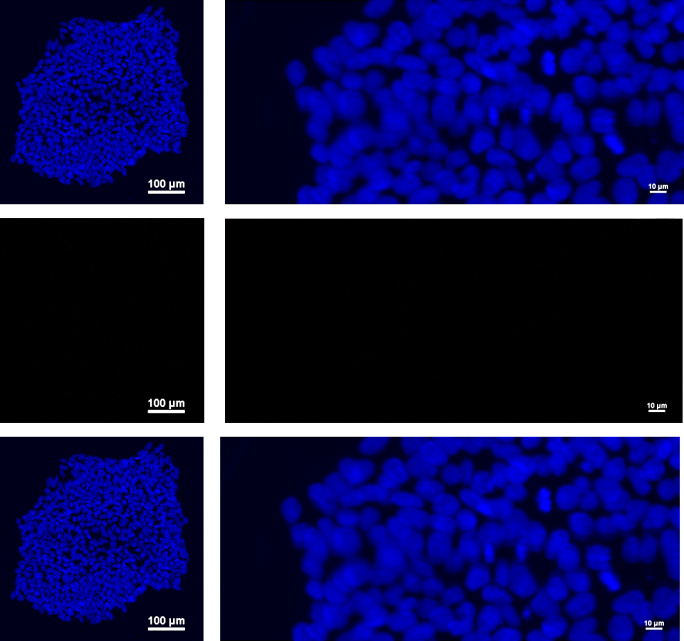

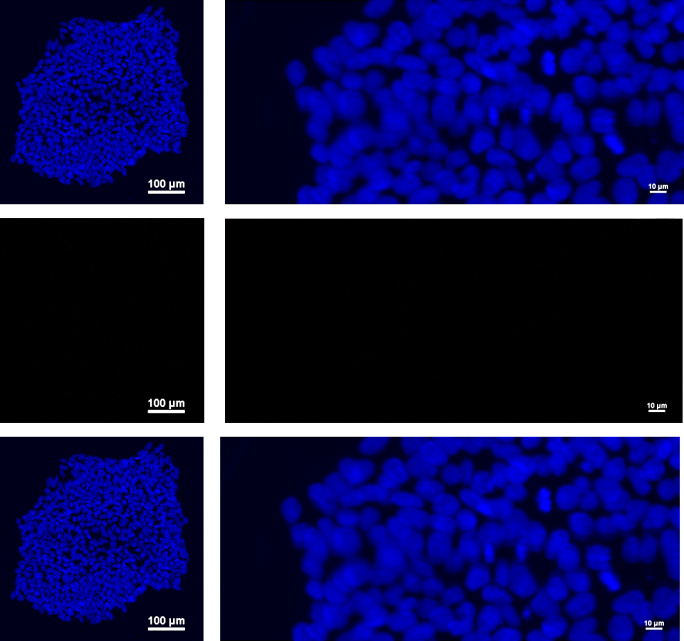

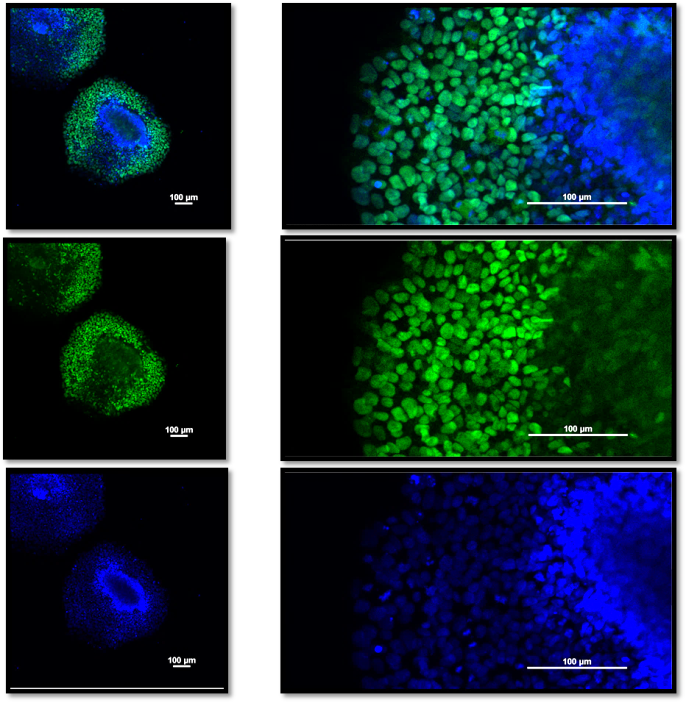


50 µm


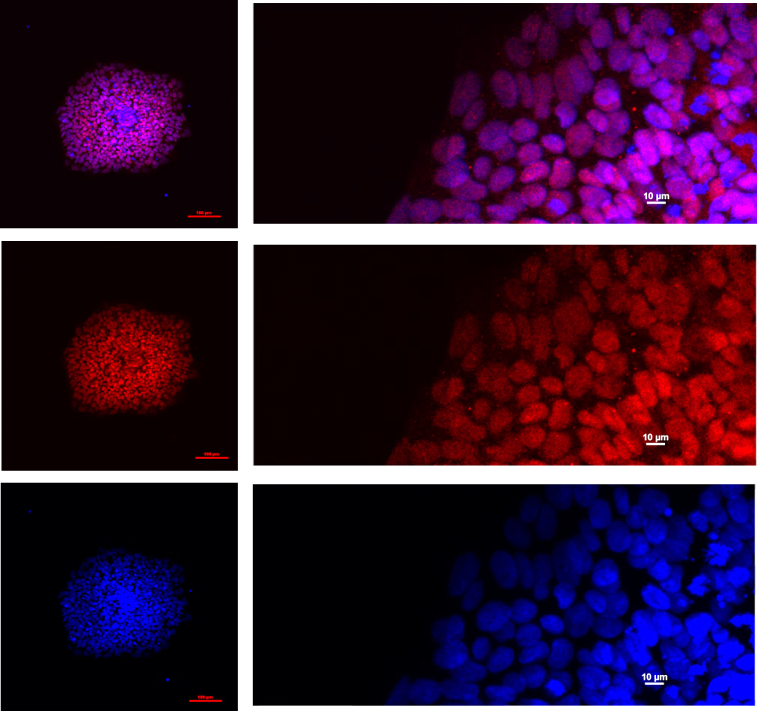


50 µm

50 µm


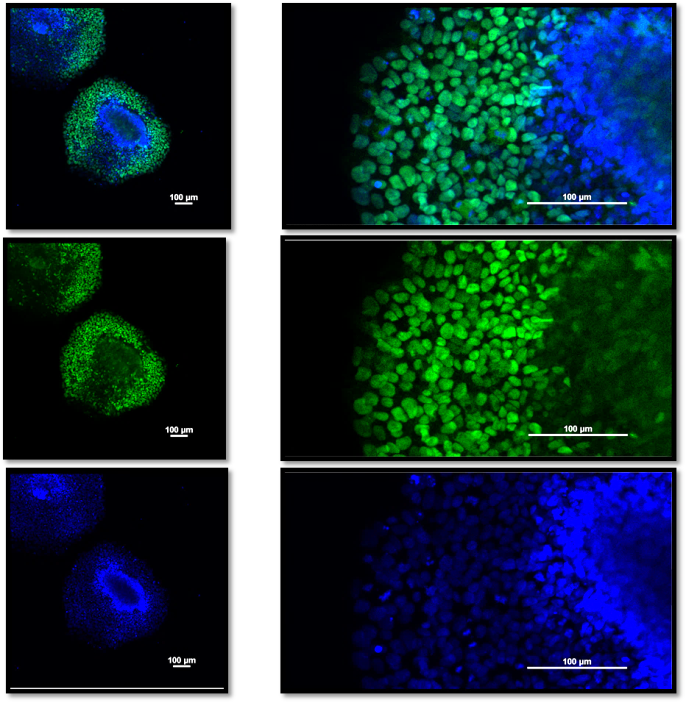


100 µm

OCT4

100 µm


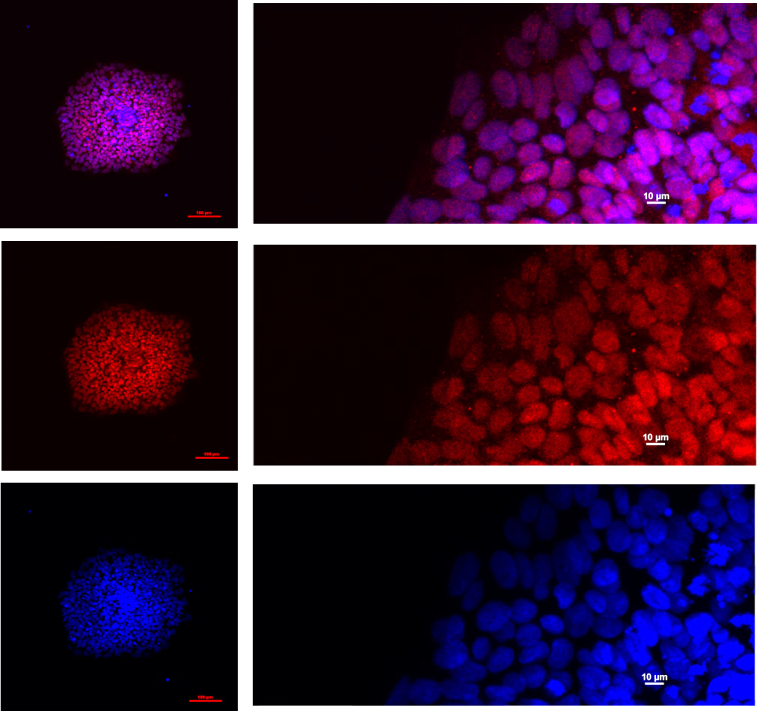


SOX2

Control


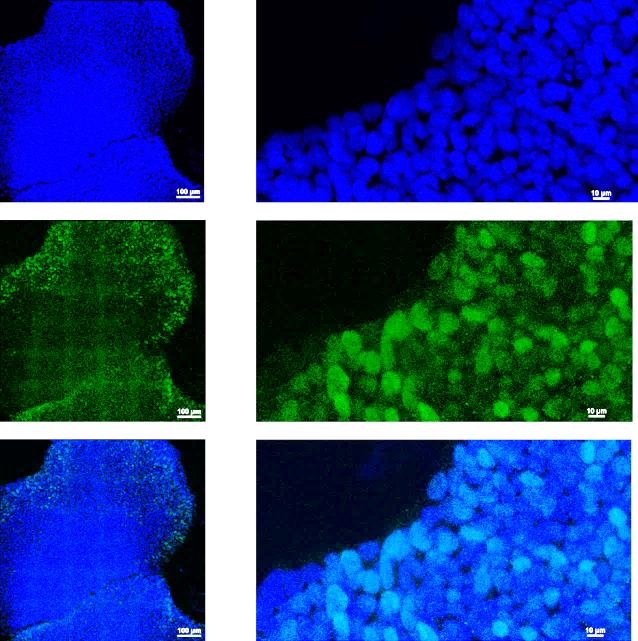

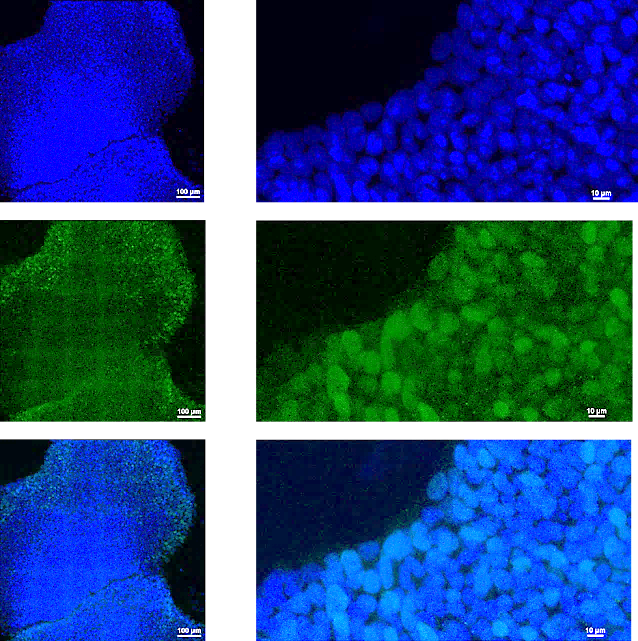


100 µm

NANOG

50 µm

100 µm

A.

**Fig. S1 ‘Positive and negative controls for OCT4, SOX2 and NANOG antibodies used in immunohistochemistry studies’.**

**(A)** Human prostate-derived induced-pluripotent stem cells were selected and utilised as positive controls for the OCT4, SOX2 and NANOG antibodies used in the immunohistochemistry studies. Immunofluorescence staining was undertaken as previously described and confirmed iPSCs to express OCT4, SOX2 and NANOG [23]. A negative ‘control’ was prepared by incubating with secondary antibody only and without primary antibody. **(B)** In the immunohistochemistry studies, a negative control was also prepared by incubating with secondary antibody only and without primary antibody.

**
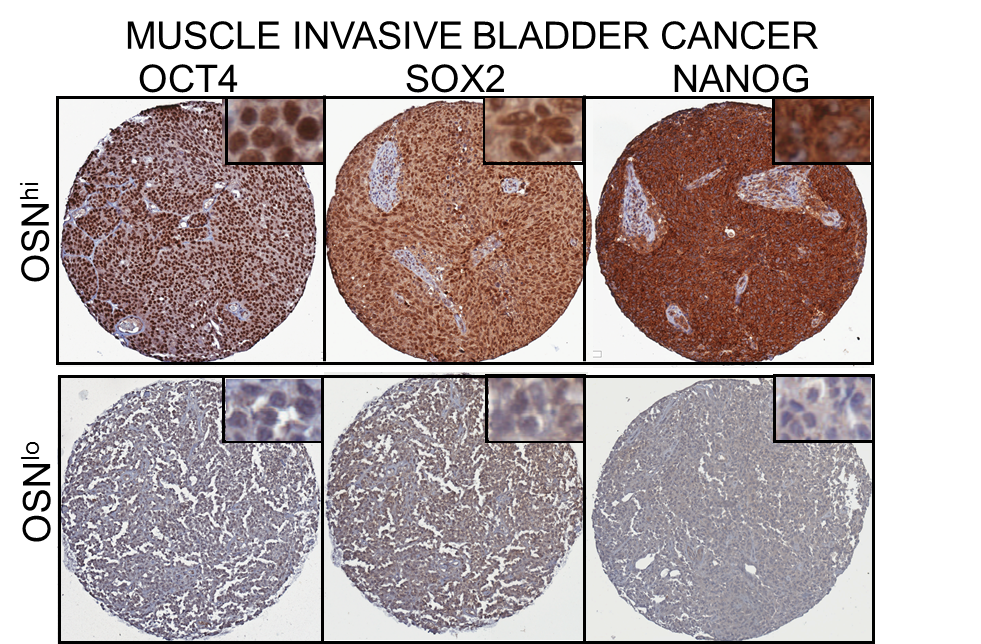
**

A.

B.

C.

**
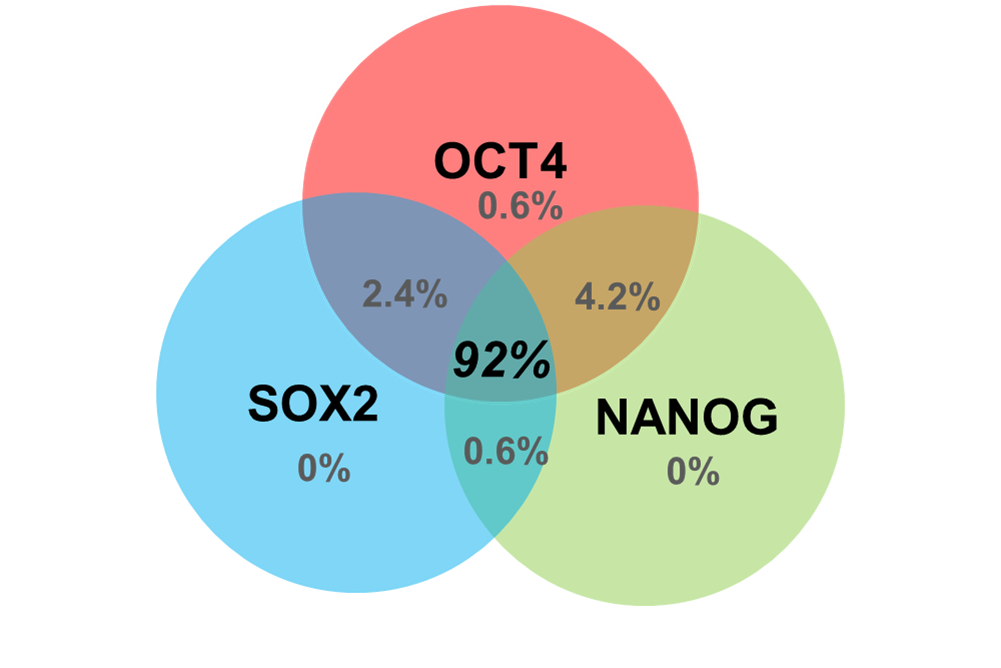

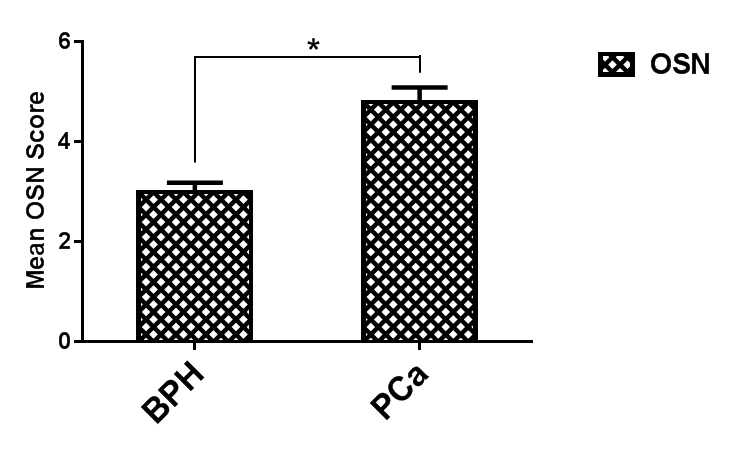
**

**
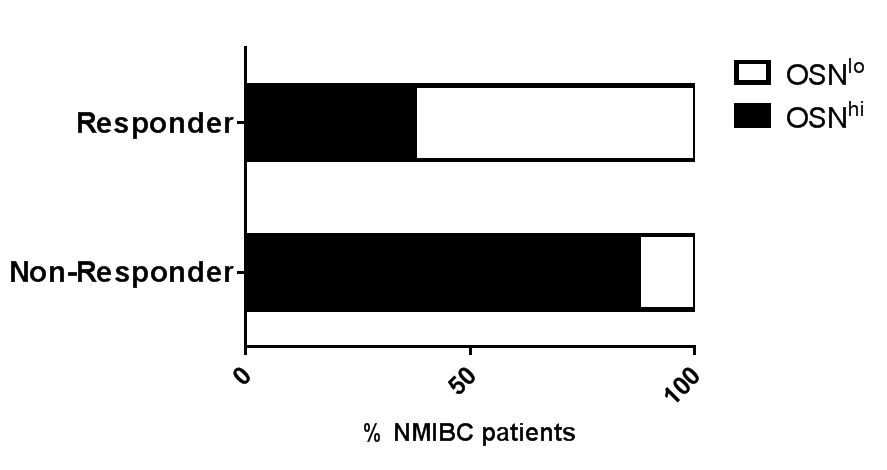
**

D.

E.

**
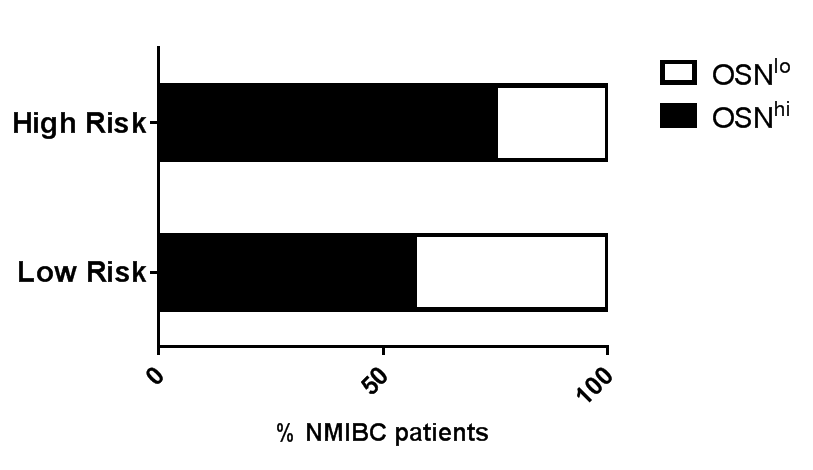
**

**
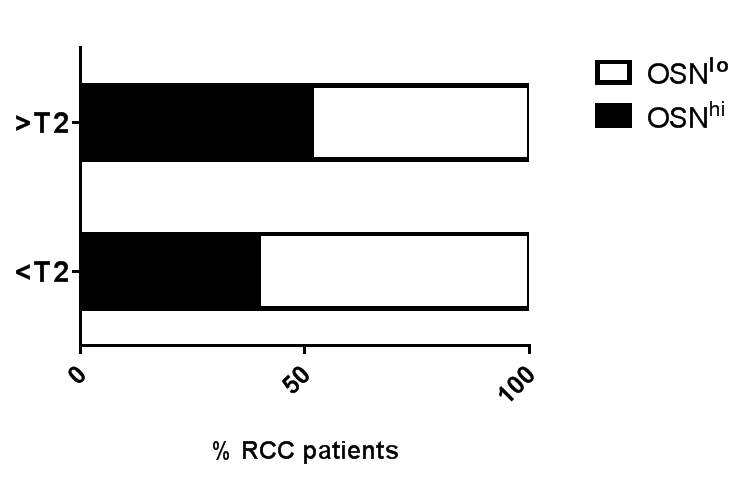
**

F.

**Fig. S2 ‘Interrogation of OSN expression signature in urological cancers’.**

**(A)** Comparison of OCT4, SOX2 and NANOG (OSN) sum score between benign prostatic hyperplasia (BPH, n=34) and prostate cancer (PCa, n=67) patients (*t-*test, *p*=0.0002).

**(B)** Venn diagram demonstrating frequency of OCT4, SOX2 and NANOG expression in non-muscle-invasive bladder cancer (NMIBC) patients.

**(C)** Illustrative tissue cores of NMIBC stained for OCT4, SOX2 and NANOG representative of OSN^hi^ and OSN^lo^ cancer patients.

**(D)** Comparison of OSN expression and risk of disease progression in NMIBC. Patients at high risk of progression to muscle invasive disease are characterised by high histological grade or early invasive histological stage into the lamina propria invasion (TNM T-stage 1). (OSN^hi^ = 110 and OSN^lo^ = 60; χ^2^-test, *p*=0.02)

**(E)** Comparison of OSN expression and response to intravesical chemotherapy in NMIBC patients (Responders = 38 and Non-Responders =14).

**(F)** Comparison of OSN expression and tumour stage (T) in renal cell cancer (RCC) patients (OSN^hi^ = 142 and OSN^lo^ = 175, χ2-test, p=0.04).

**
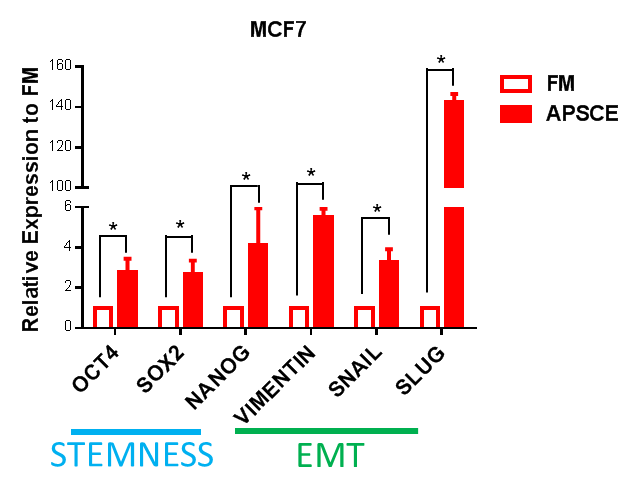

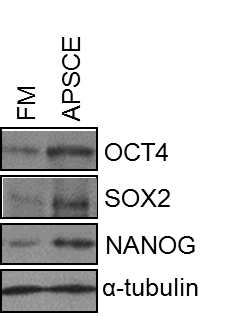
**

A.

**
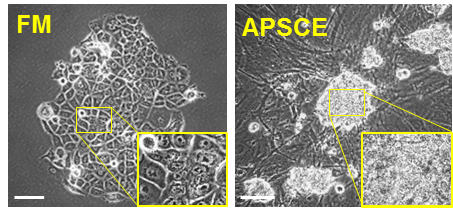
**

**
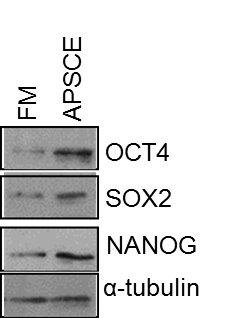
**

**
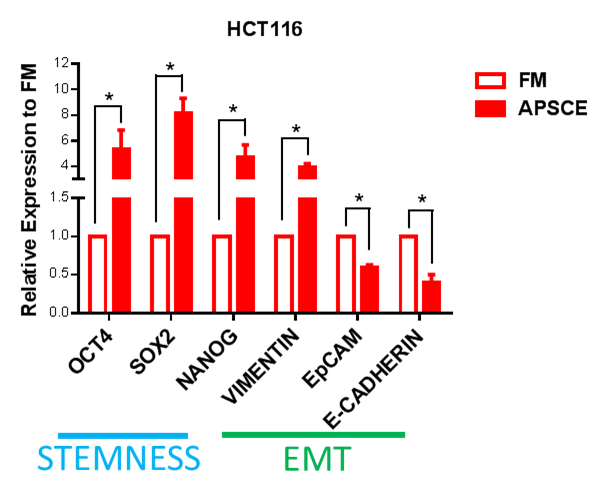
**

B.

**
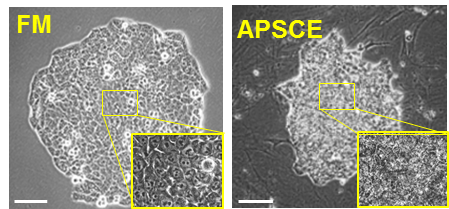
**

**
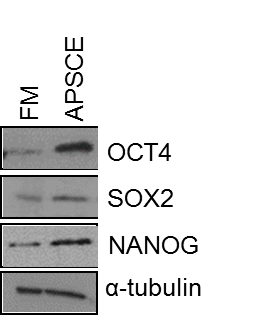

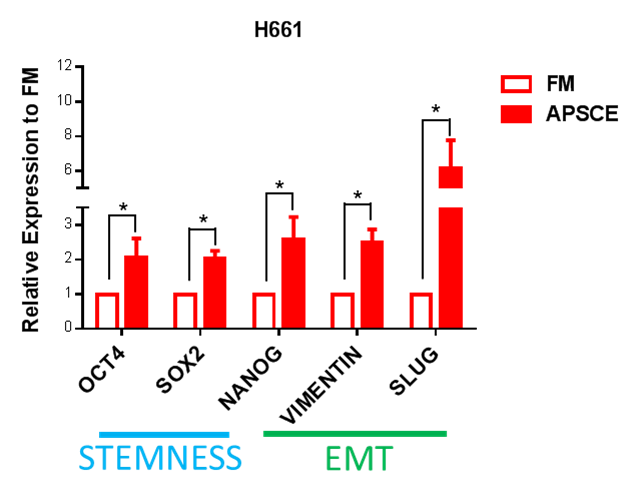
**

C.

**
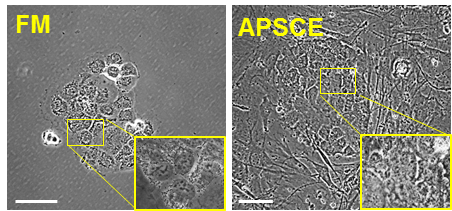
**

**Fig. S3 ‘Establishment of APSCE model in multiple human epithelial cancer cell lines’.**

**(A)** Stemness and mesenchymal gene expression by qPCR (left panel), OSN expression by western blotting (middle panel) and cell growth after 7 days (right panel) was measured following culture in serum supplemented medium (FM) vs stem cell environment (APSCE) in breast cancer cells (MCF7). Data represents at least three independent experiments ± SEM. (*denotes *p*-value < 0.05). Scale bar 10µm. Of note, EMT characterisation studies were carried out without feeders in APSCE. For cells cultured in APSCE in the morphology studies, feeder cells were used though no MACS selection was performed. These approaches were undertaken to avoid any concerns about effects due to EMT induction and any mesenchymal cell contamination.

**(B)** Same as in (A) but in colon cancer cells (HTC116).

**(C)** Same as in (A) but in lung cancer cells (H661).

A.

**
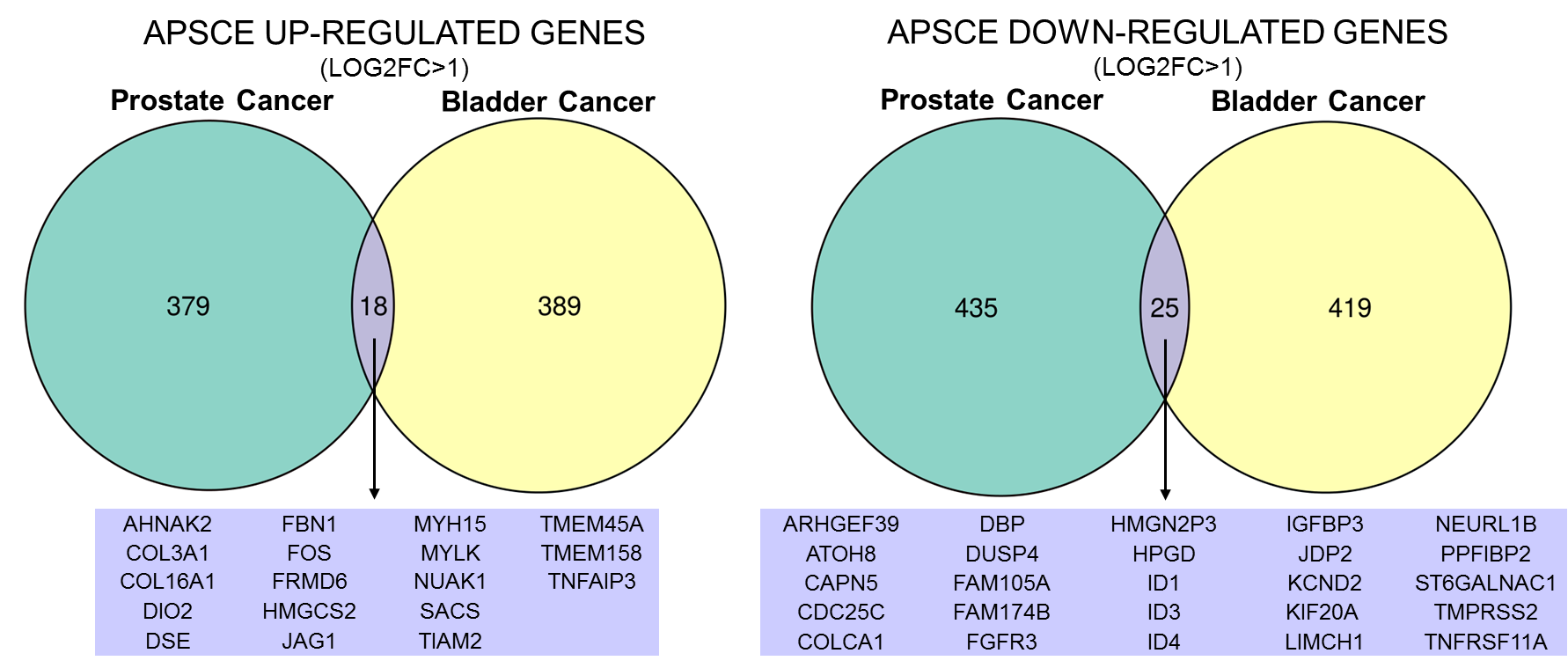
**

B.

**
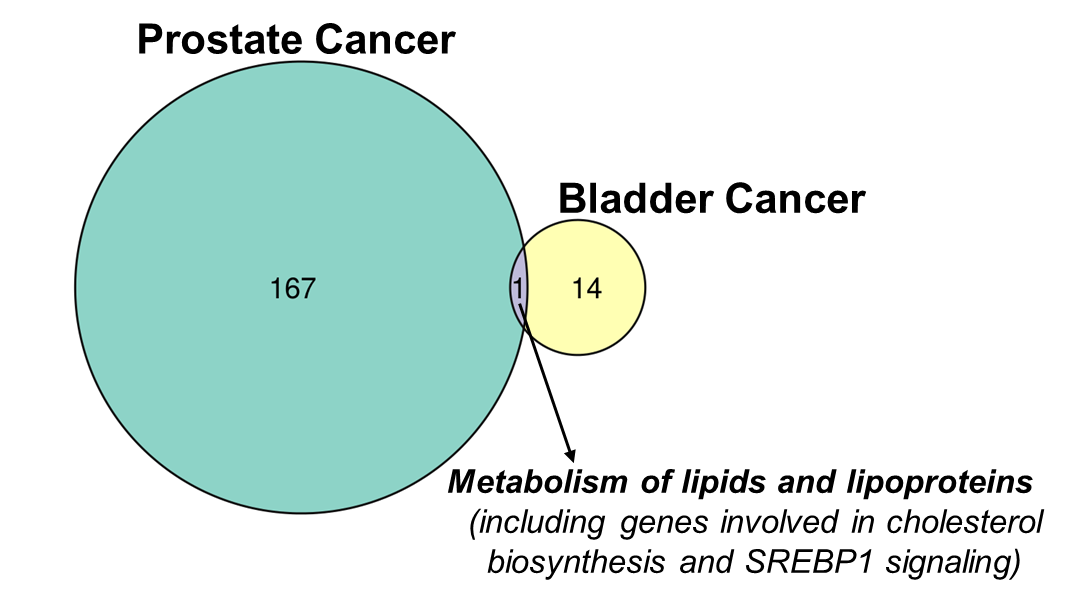
**

**Fig. S4 ‘RNA sequencing analysis of prostate and bladder cancer cells in APSCE’.**

**(A**) Venn diagrams of genes demonstrating ≥2-fold up- and down-regulation following culture in APSCE in comparison to culture in FM in prostate (LNCaP) and bladder (RT112) cancer cells as analysed by RNA sequencing of three separate biological experiments. The overlapping up- and down-regulated genes in prostate and bladder cancer cells are noted.

**(B**) Venn diagram demonstrating common pathways in APSCE for prostate (LNCaP) and bladder cancer (RT112) cells. The ‘metabolism of lipids and lipoproteins (including genes involved in cholesterol biosynthesis and SREBP1 signalling’ pathway was identified.


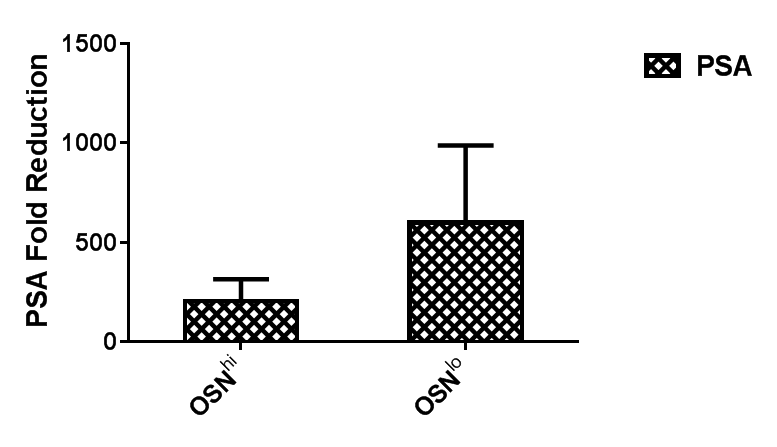


A.

B.

C.

D.


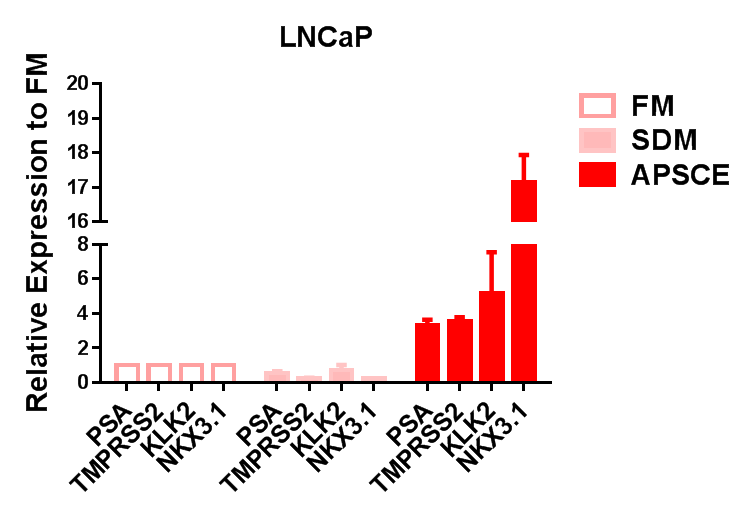

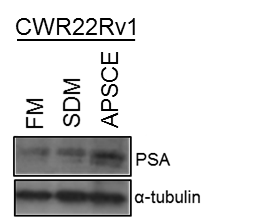

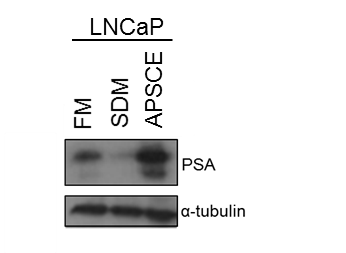


**Fig. S5 ‘Induction of OSN and androgen-regulated genes in APSCE in prostate cancer cells’.**

**(A)** Comparison of fold reduction in PSA (PSA at presentation/PSA nadir) between OSN^hi^ and OSN^lo^ prostate cancer patients.

**(B)** Western blot analysis measuring PSA protein expression in CWR22Rv1 cells cultured in FM, steroid depleted medium (SDM) and APSCE for 24h. α-tubulin was used as loading control.

**(C)** Expression of *PSA, KLK2, TMPRSS2* and *NKX3.1* was measured by qPCR in LNCaP cells cultured in FM, SDM and APSCE. Data is represented as fold change of FM experimental arm. Data represents at least three independent experiments ± SEM.

**(D)** Same as in (B) but in LNCaP cells.

**
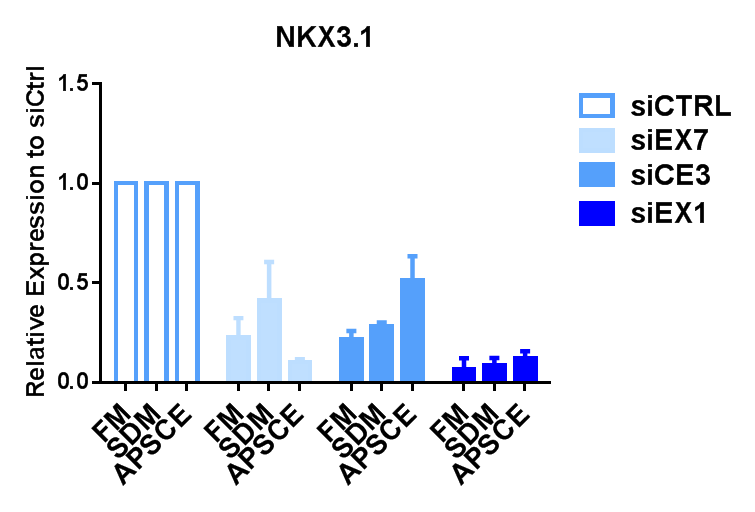

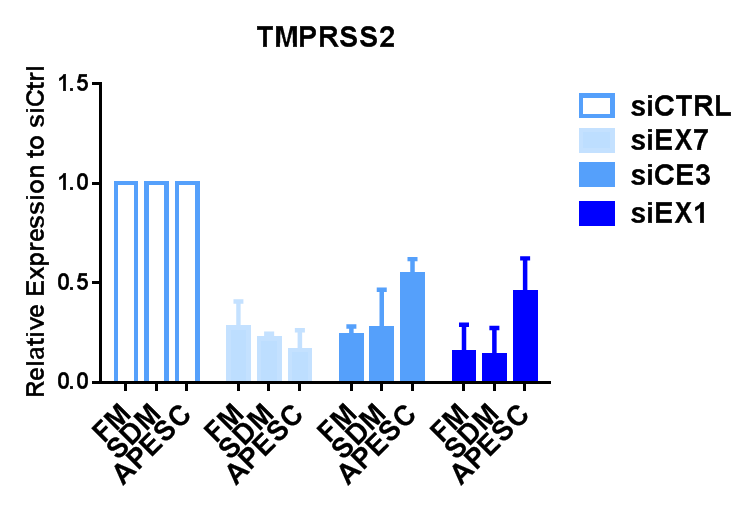
**

A.

B.

C.

D.

**
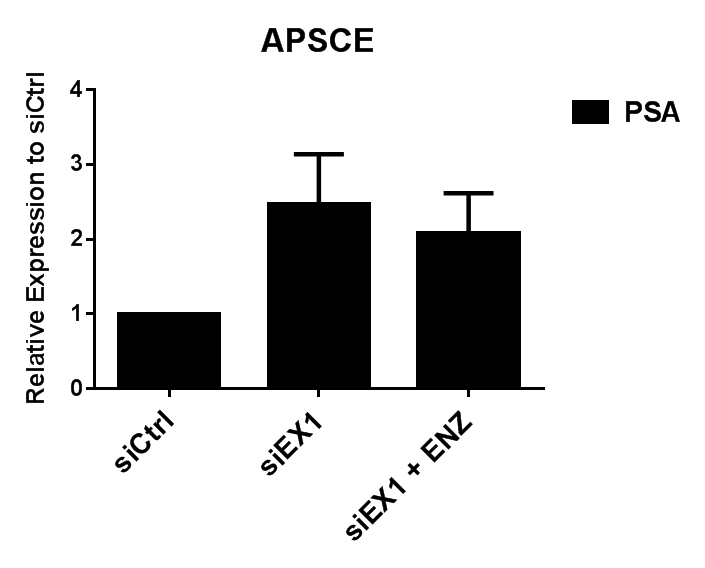

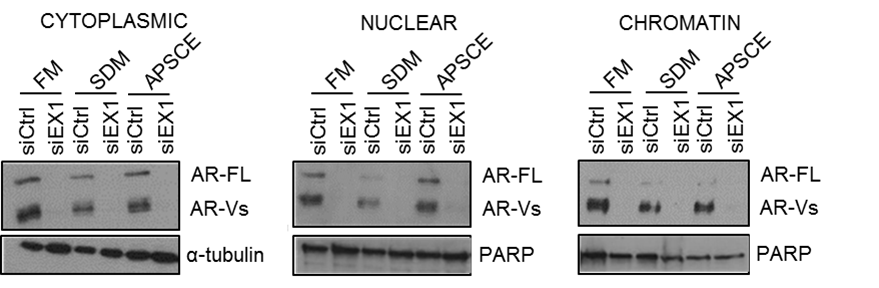
**

**Fig. S6 ‘Androgen-regulated gene expression following depletion of AR in APSCE in CWR22Rv1 cells’.**

**(A)** Expression of NKX3.1 was measured by qPCR following knockdown of AR-FL (siEX7), AR-V7 (siCE3) and all AR (siEX1) in CWR22Rv1 cells cultured in FM, SDM or APSCE. Data is represented as fold change of siCTRL experimental arm. Data represents at least three independent experiments ± SEM.

**(B)** Same as in (A) but for TMPRSS2 expression.

**(C)** Western blot analysis demonstrating localisation of AR-FL and AR-Vs following isolation of cytoplasmic (left panel), nuclear (middle panel) and chromatin (right panel) fractions of CWR22Rv1 cells transiently transfected with siCTRL and siEX1 for 48h and followed by culture in FM, SDM and APSCE. α-tubulin and PARP were used as cytoplasmic and nuclear loading controls, respectively.

**(D)** Effect of treatment with 10 µM antiandrogen enzalutamide (ENZ) on PSA expression in CWR22Rv1 cells transfected with siEX1. Data is represented as fold change of siCTRL experimental arm. Data represents at least three independent experiments ± SEM.

**
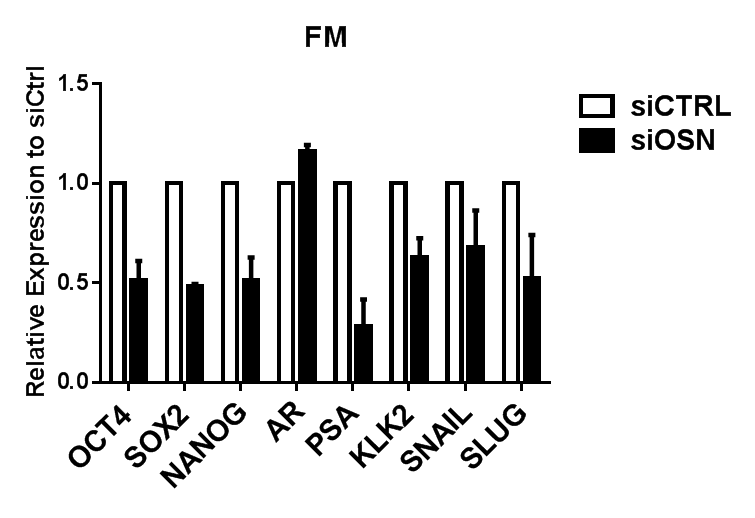

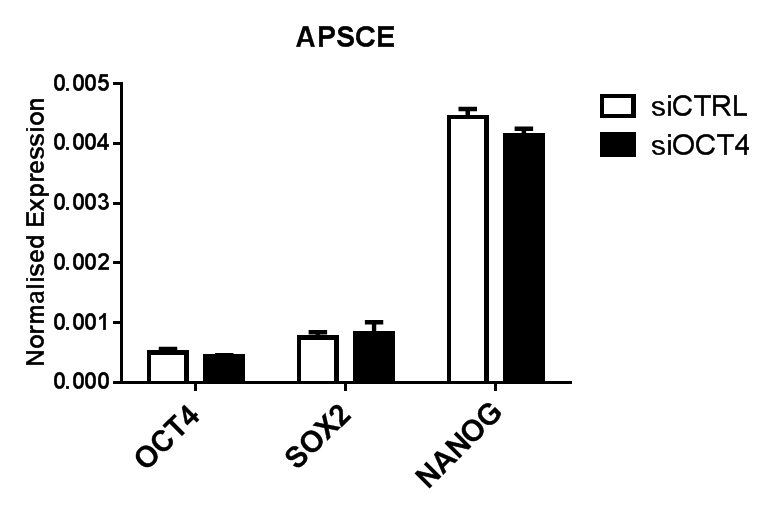
**

A.

B.

**
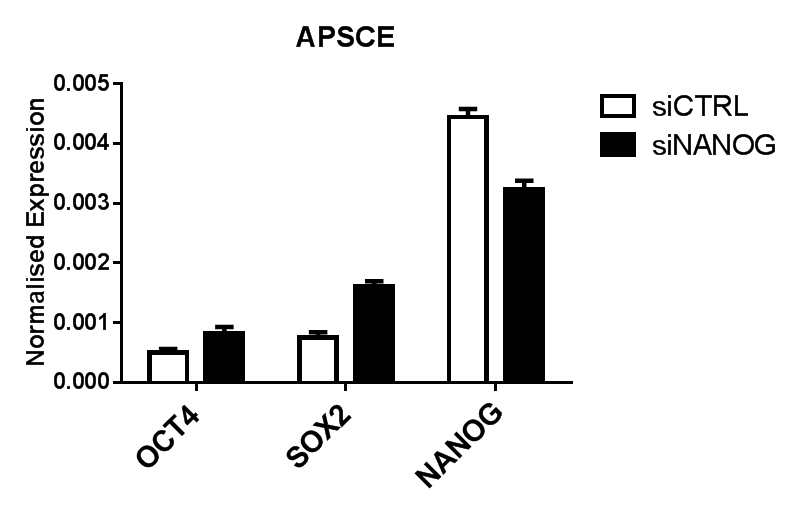

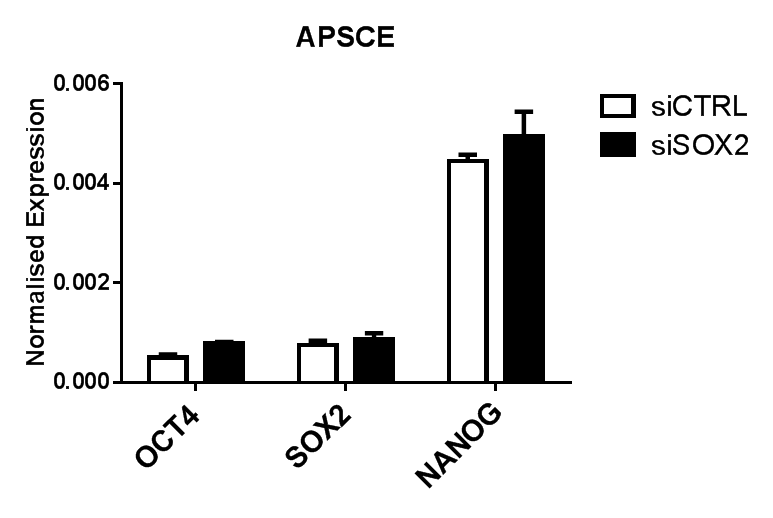
**

D.

C.

**Fig. S7 ‘Triple knockdown for OCT4, SOX2 and NANOG was achieved in FM but was not possible in APSCE’.**

**(A)** Expression of OCT4, SOX2, NANOG, AR, PSA, KLK2, SNAIL and SLUG was measured by qPCR following triple knockdown of OCT4, SOX2 and NANOG (siOSN) in CWR22Rv1 cells cultured in FM. Data is represented as fold change of siCTRL experimental arm. Data represents at least three independent experiments ± SEM.

(**B**) Expression of OCT4, SOX2 and NANOG following knockdown of OCT4 (siOCT4) in CWR22Rv1 cells cultured in APSCE.

**(C)** Same as in (B) but following knockdown of SOX2.

**(D)** Same as in (C) but following knockdown of NANOG.

**
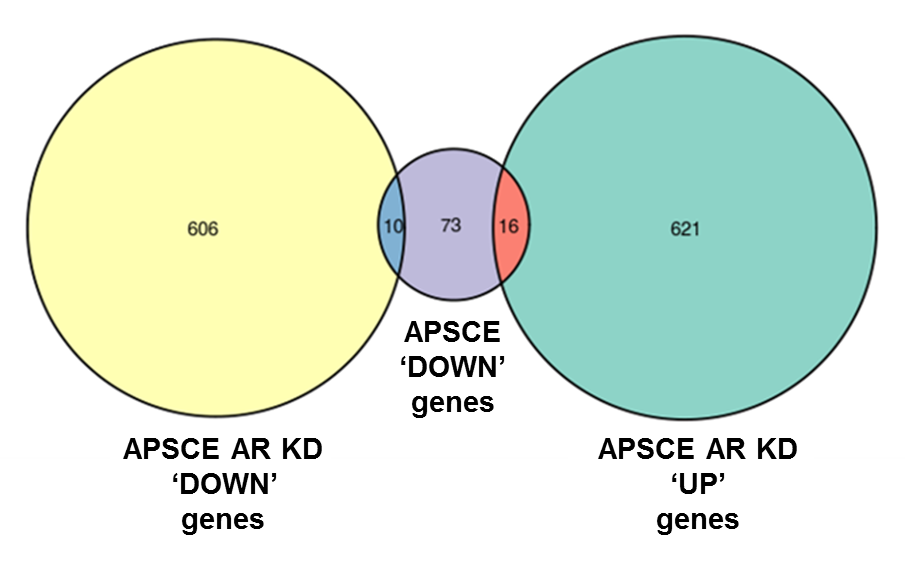

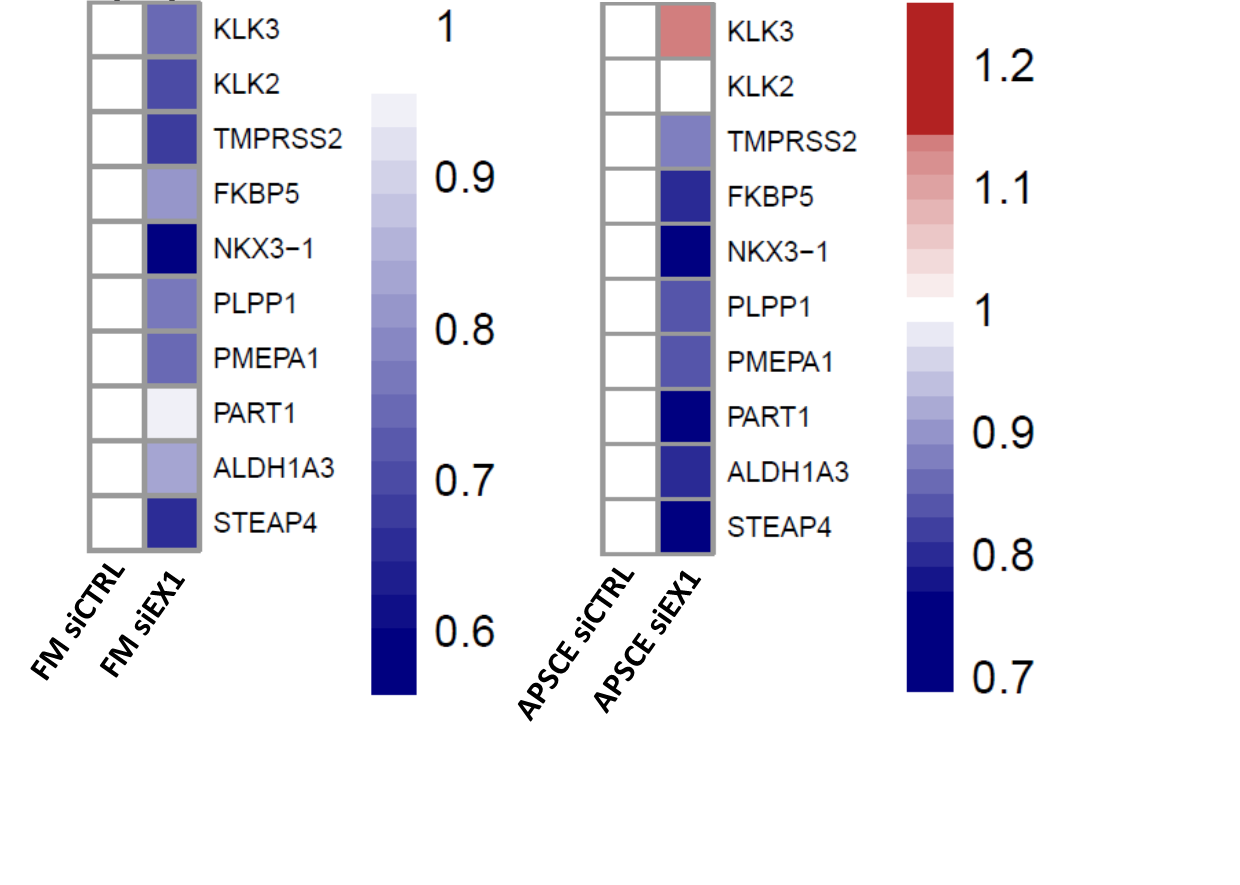
**

B.

A.

**Fig. S8 ‘Gene expression in APSCE following knockdown with siEX1 as determined from RNA-sequencing analysis’.**

**(A)** Heat maps of androgen-regulated gene expression in FM and APSCE following knockdown with siEX1 in CWR22Rv1 cells determined from RNA-sequencing. Data represents the average normalised read counts of three independent experiments Log2 transformed and relative to respective siCTRL.

**(B)** Venn diagram demonstrating genes downregulated in APSCE and overlapping up- or down-regulated following AR depletion with siEX1 in APSCE in CWR22Rv1 cells.


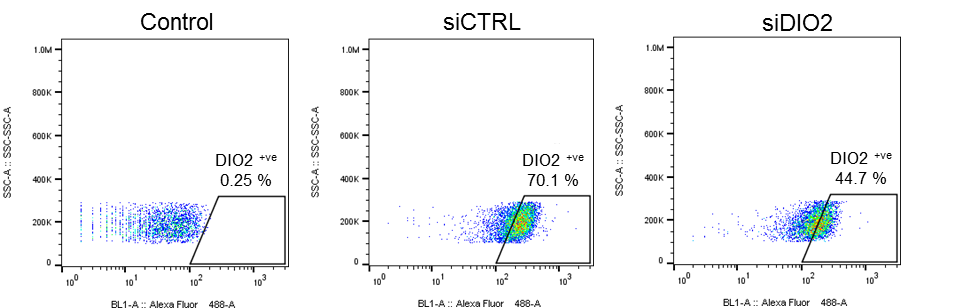


A.

C.

B.


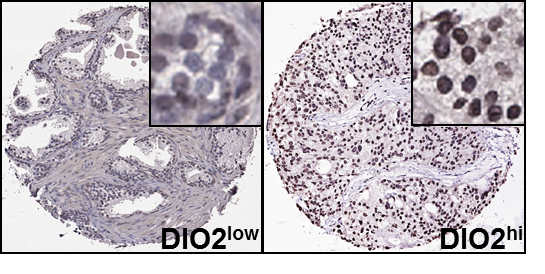
**
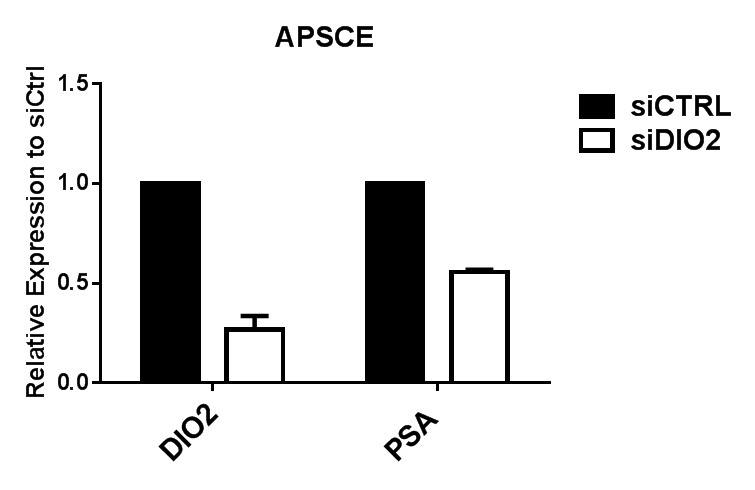
**


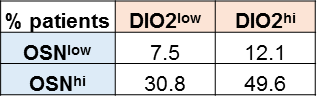


**Fig. S9 ‘DIO2 expression in prostate cancer cells’.**

**(A)** Expression of DIO2 following knockdown with siDIO2 in APSCE in CWR22Rv1 cells was determined by flow cytometry.

**(B)** Expression of DIO2 and PSA following knockdown with siDIO2 in APSCE in CWR22Rv1 cells was measured by qPCR. Data is represented as fold change of siCTRL experimental arm. The reduction in PSA expression was comparable to the maximal 40-50% protein knockdown in DIO2 expression that was achievable in (A).

**(C)** Tissue expression of DIO2 demonstrates utility across prostate cancer cohort based on hormone treated prostate cancer patients (n=133, left panel). Examples of low (DIO2^low^) and high (DIO2^hi^) levels of DIO2 expression in prostate cancer patients (right panel).

**Supplementary Tables**

**Table S1. Demographic characteristics of prostate cancer patients.**

**
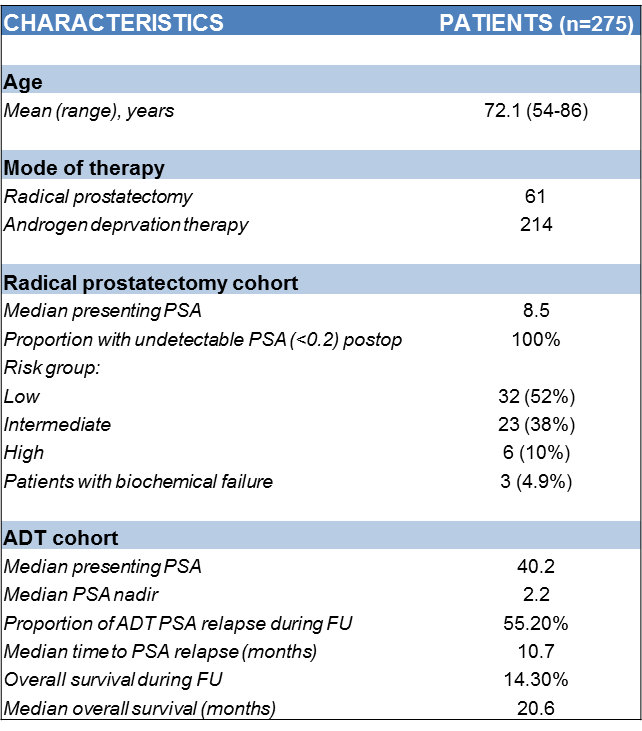
**

FU: Follow up.

**
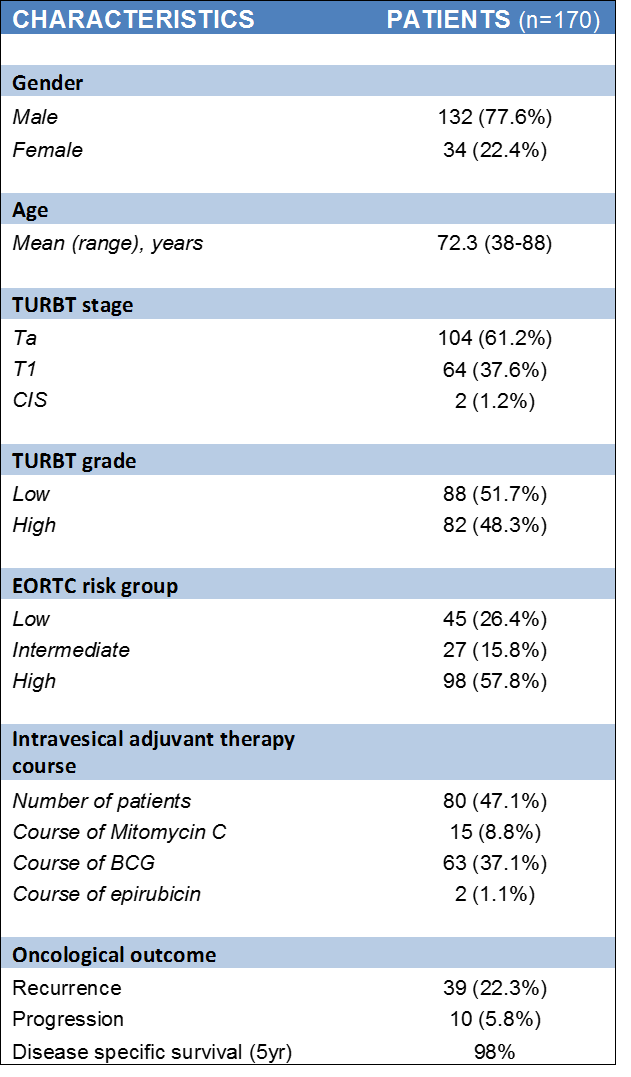
Table S2. Demographic characteristics of NMIBC cancer patients.**

TURBT: Trans urethral resection of bladder tumour, EORTC: European organisation for research and treatment of cancer, BCG: Bacillus Calmette-Guerin.

**
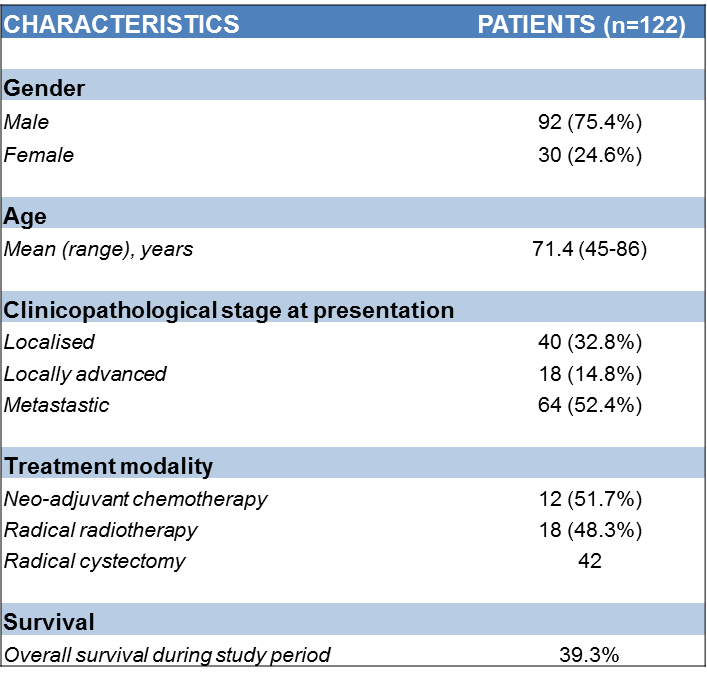
Table S3. Demographic characteristics of MIBC cancer patients.**

**Table S4. Demographic characteristics of renal cancer patients.**

**
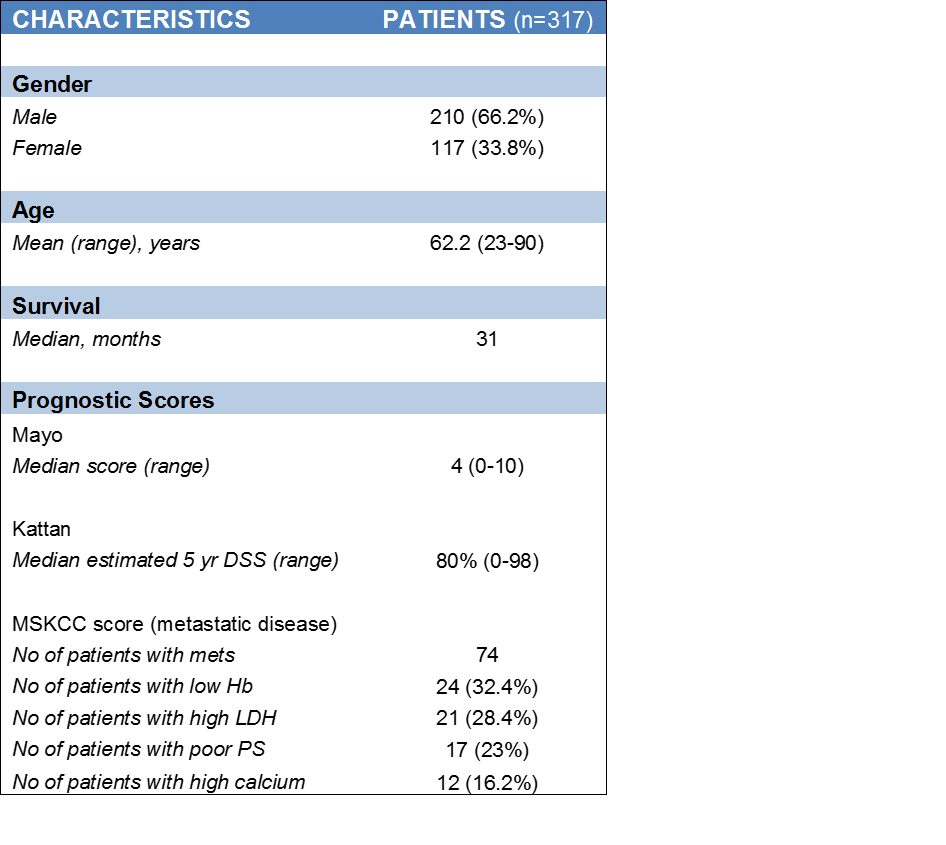
**

DSS: Disease Specific Survival, Mets: Metastasis, Hb: Haemoglobin, LDH: Lactate dehydrogenase, PS: Performance status.

**Table S5. Mass transitions and optimised MS/MS parameters**


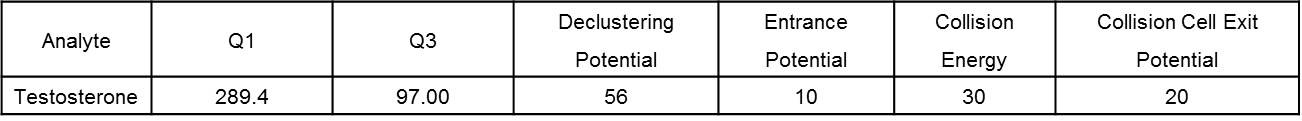


**Table S6. Testosterone assay parameters (^*^ defined as 3 x baseline signal, ^**^ defined as 10 x baseline signal, ^†^ in ng/ml, ^ᵞ^ Square of the Pearson Product Moment Correlation)**


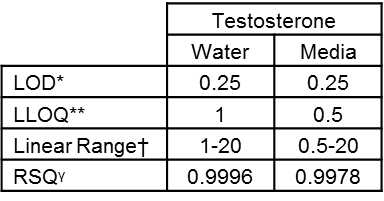


**Table S7. Chromatographic conditions**


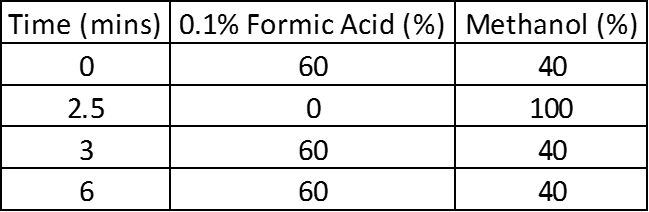


**Table S8. Mass spectrometer Ion Source settings**


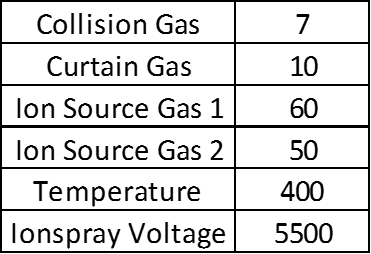


**Table S9. List of primers for real-time reverse transcription-polymerase chain reaction**


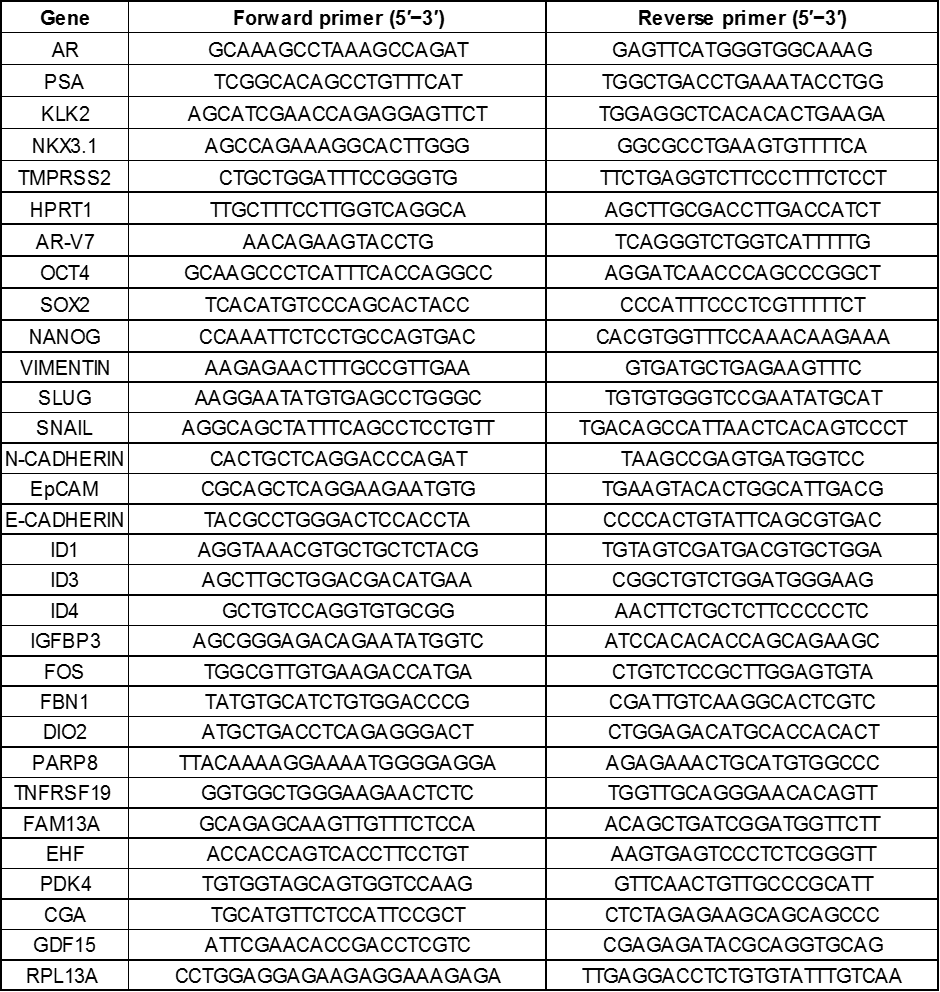

Supplement: Supplementary file 1 — SI DATA [file 41388_2019_712_MOESM1_ESM.docx]
